# Transcriptome profiling reveals insertional mutagenesis suppressed the expression of candidate pathogenicity genes in honeybee fungal pathogen, *Ascosphaera apis*

**DOI:** 10.1038/s41598-020-64022-3

**Published:** 2020-05-05

**Authors:** Awraris Getachew, Tessema Aynalem Abejew, Jiangli Wu, Jin Xu, Huimin Yu, Jing Tan, Pengjie Wu, Yangyang Tu, Weipeng Kang, Zheng Wang, Shufa Xu

**Affiliations:** 1 Key Laboratory of Pollinating Insect Biology, Ministry of Agriculture; Institute of Apicultural Research, Chinese Academy of Agricultural Sciences, 100093 Beijing, China; 2 College of Agriculture and Environmental Sciences, Bahir Dar University, Bahir Dar, Ethiopia

**Keywords:** Computational biology and bioinformatics, Molecular biology

## Abstract

Chalkbrood disease is caused by *Ascosphaera apis* which severely affects honeybee brood. Spore inoculation experiments shown pathogenicity varies among different strains and mutants, however, the molecular mechanism of pathogenicity is unclear. We sequenced, assembled and annotated the transcriptomes of wild type (SPE1) and three mutants (SPE2, SPE3 and SPE4) with reduced pathogenicity that were constructed in our previous study. Illumina sequencing generated a total of 394,910,604 clean reads and *de novo* Trinity-based assembled into 12,989 unigenes, among these, 9,598 genes were successfully annotated to known proteins in UniProt database. A total of 172, 3,996, and 650 genes were up-regulated and 4,403, 2,845, and 3,016 genes were down-regulated between SPE2-SPE1, SPE3-SPE1, and SPE4-SPE1, respectively. Overall, several genes with a potential role in fungal pathogenicity were detected down-regulated in mutants including 100 hydrolytic enzymes, 117 transcriptional factors, and 47 cell wall related genes. KEGG pathway enrichment analysis reveals 216 genes involved in nine pathways were down-regulated in mutants compared to wild type. The down-regulation of more pathways involved in pathogenicity in SPE2 and SPE4 than SPE3 supports their lower pathogenicity during *in-vitro* bioassay experiment. Expression of 12 down-regulated genes in mutants was validated by quantitative real time PCR. This study provides valuable information on transcriptome variation caused by mutation for further functional validation of candidate pathogenicity genes in *A. apis*.

## Introduction

Honeybees face serious challenges from pathogens, among these, *Ascosphaera apis* is an entomopathogenic filamentous fungus that causes chalkbrood disease in honeybees which exclusively affects honeybee brood^1,2^. Subsequently, chalkbrood disease reported to reduce 5–37% of honey production and to cause 12–92% brood death^3–5^. Several pathogenicity experiments have been carried out using artificial methods, such as spore inoculation and *in vitro* larval rearing techniques^6^ which reported to vary between different strains^5^, and between wild-type and mutants^7,8^. Random mutant library construction is a useful method to identify genes of unknown functions in microorganisms^9^. In previous study, we constructed mutants from the wild-type strain of *A. apis* using Restriction Enzyme-Mediated Integration (REMI) technique and we obtained less pathogenic and nonpathogenic mutants in comparison to the wild type^8^. However, the result of pathogenicity assay could not clearly justify what happened to less pathogenic mutants at molecular level.

All the cells of an organism share similar genetic code and the proper regulation of gene expression is among the key processes that confers cell identity by activating a specific subset of genes in a given cell type. Therefore, understanding gene regulation is vital in unravelling the effect of genetic variation on both normal development and mutations. The best approach to uncover the process which enables a fungus to be pathogenic and colonizes a honeybee larva is identifying the genes and understanding the molecular mechanism that contribute to pathogenicity.

Transcriptome analysis primarily focuses on individual gene expression changes in affected versus unaffected individuals but also allows to understand the coordinated function of multiple genes by looking at co-expression networks^10^. The reason behind is that co-expression expected to reflect genes that belong to common regulatory pathways^11^. Currently, RNA-seq technology has emerged as a cost-effective approach in high-throughput sequence determination for the discovery of functional genes faster. In this study, we sequenced mRNA from hyphae and spore of *A. apis* using Illumina sequencing. Furthermore, orthologous transcripts have been identified and compared to confirm those differentially or uniquely expressed in wild-type (SPE1) and the three mutants (SPE2, SPE3, and SPE4). Several genes associated with fungal pathogenesis, the KEGG pathway and transcription factors were differentially expressed among mutants and wild type strains of *A. apis*.

However, analyzing the experiments of gene expression classically produces abundant differentially expressed genes (DEGs), without considering for possible sophisticated mechanisms of interactions^12,13^. The results of such DEGs are better interpreted by analysis of biological pathways rather than analysis of those interacted genes individually^14^. Pathway enrichment analysis reported to be the primary research for understanding insight into the innate mechanism of DEGs^15^, to distinguish the related pathways that significantly enriched between two experimental conditions^16^. Furthermore, confirmation of protein-protein interactions (PPIs) is important for researching molecular activity involved in living cell by learning how proteins work together in a harmonized manner to perform cellular functions. However, to the best of our knowledge, pathways changed due to insertional mutagenesis and PPIs of *A. apis* have not yet been investigated. We aimed to identify the pathogenic genes and screen the potential pathways changed and associated protein-protein interaction networks in *A. apis* mutants in comparison to wild type.

## Results

### Sequencing and transcriptome *de novo* assembly

Sample RNA was prepared from hyphae and spore of three mutants and their original wild-type. Illumina mRNA sequencing was performed for three biological replicates of each sample: wild-type SPE1 (SPE1–1, SPE1–2 and SPE1–3), mutant SPE2 (SPE2–1, SPE2–2 and SPE2–3), mutant SPE3 (SPE3–1, SPE3–2 and SPE3–3) and mutant SPE4 (SPE4–1, SPE4–2 and SPE4–3). Sequencing data of the RNA-seq samples are shown in Table 1. For SPE1, about 33 million clean reads comprising 6 billion nucleotides were produced (Table 1). For each of the mutant of SPE2, SPE3 and SPE4 approximately 29, 36 and 32 million clean reads, containing a total of 5.2, 6.8 and 5.8 billion nucleotides, respectively, were obtained (Table 1). Sequence data totaling 37.8 Gbases has been deposited in the NCBI with accession number SRR9021798–9021809.

**Table 1:** Summary of sequences analysis.

Sample	Raw reads	Clean reads	Mapped reads	Percentage of total reads	Clean bases (Gb)	Q20 (%)	Q30 (%)	GC (%)
SPE1–1	48349304	37105430	26438255	54.68	7.25	99.95	99.6	40.5
SPE1–2	41845696	30077548	21464648	51.29	6.28	99.95	99.55	40.5
SPE1–3	40884398	31540444	22643882	55.39	6.13	99.95	99.65	40.5
SPE2–1	35598878	28872174	20453258	57.45	5.34	99.95	99.7	39.5
SPE2–2	35768122	29357398	20893501	58.41	5.37	99.95	99.7	39.5
SPE2–3	34363944	28938056	20503537	59.67	5.15	99.95	99.7	39.5
SPE3–1	47172926	38797772	27184234	57.63	7.08	99.9	99.55	40.5
SPE3–2	46434824	37086102	26465041	56.99	6.97	99.95	99.8	40.5
SPE3–3	42874686	34390720	24481896	57.10	6.43	99.95	99.7	40
SPE4–1	41120690	34729034	24769384	60.24	6.17	99.95	99.7	42
SPE4–2	38580564	32795348	23255301	60.28	5.79	99.95	99.75	42
SPE4–3	37590610	31220578	22272348	59.25	5.64	99.95	99.7	42
Summary	490584642	394910604			73.6			

All transcriptome reads (394,910,604) pooled from the 12 samples were employed in the *de novo* assembly (Table 2). A total of 284,718 transcripts have been assembled with an N50 length of 2,334 bp, an average transcript length of 1,207.469275 bp and a maximum transcript length of 17,892 bp by the trinity method (Table 2). A total of 12,989 unigenes were predicted accordingly and the length of a unigenes was raged from201 to 5077 bp with an average size of 758.9073832 bp (Table 2). BUSCO analysis showed that 100% of the transcripts were complete (C:100.0% [S: 7.9%, D: 92.1%], F: 0.0%, M: 0.0%, n: 290). Furthermore, PCA analysis revealed the mutant samples clearly separated from wild-type, indicating a visible variation among the different samples (Supplementary information, Fig. S1).

**Table 2:** Statistics of transcriptome assembly and predicted unigenes.

Variable	Trinity	Corset
Number	284718	12989
Size of data (bp)	343788237	9857448
Minimum length (bp)	201	201
Maximum length (bp)	17892	5077
Mean length (bp)	1207.469	758.9074
N50 length (bp)	2334	853
GC content	43.24	55.58

### Functional annotation

Identification of the putative functions of *A. apis* unigenes was performed using a BLASTx search (version 2.8.0 + ). Of the 12,989 unigenes, 73.89% (9,598) were successfully identified as known proteins in UniProt database. In the other five databases (InterPro, Pfam, GO, KEGG, eggnog), 7,807 (60.10%), 6,561 (50.51%), 6,583 (50.68%), 2503 (19.27%), and 230 (1.77%) unigenes have been confirmed as annotated proteins, respectively. While, the remaining 26.11% (3,391 unigenes), without significant identity to any sequences.

Gene Ontology analysis was further employed for unigenes of *A. apis*. To compare the molecular characterizations among mutants and to describe their associated biological processes, cellular locations and molecular functions in a mutant^17^. The category of molecular function consisted of a total of 945 GO terms, which included 12,131 unigenes while the biological process category consisted of 1,182 GO terms (8,686 unigenes) and the cellular location category had 394 GO terms (7,190 unigenes). Among the categories membrane, integral component of membrane, oxidation-reduction process, nucleus and ATP binding were the largest five subcategories (total 60 subcategories). Under the Biological Process classification, oxidation-reduction process (748 unigenes), transmembrane transport (478 unigenes), metabolic process (410 unigenes), and regulation of transcription (383 unigenes) were most significantly enriched, which showed that the related unigenes play key role in metabolism in *A. apis*. Within the Cellular Component categories, the unigenes were mainly represented to membrane (1,919 unigenes), integral component of membrane (1,853 unigenes), nucleus (726 unigenes), and cytoplasm (314 unigenes). While involved in Molecular Function classification, the main unigenes were confirmed to ATP binding (615 unigenes), hydrolase activity (592 unigenes), metal ion binding (587 unigenes), and transferase activity (560 unigenes) prominently. Out of the 12,989 unigenes, 230 (1.77%) were classified and identified in 23 functional categories (Supplementary information, Fig. S2). The “general function prediction only” category (36, 15.65%) was the most important group. The category of posttranslational modification, protein turnover, chaperones (20, 8.70%), translation, ribosomal structure and biogenesis (19, 8.26%), signal transduction mechanisms (17, 7.39%), and intracellular trafficking, secretion, and vesicular transport (17, 7.39%), were annotated by COG classification. However, only few unigenes were confirmed as extracellular structures (2, 0.87%), coenzyme transport and metabolism (2, 0.87%), and nuclear structures (1, 0.43%).

### KEGG pathway analysis

**Figure 1:**
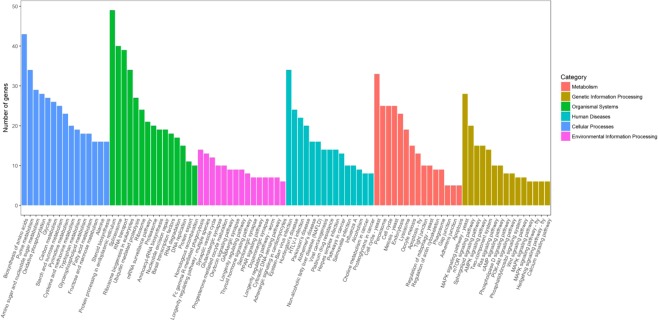
KEGG categories mapped from the annotated proteins. The vertical axis lists the names of pathways in the KEGG database, and the horizontal axis shows the proportion of annotated genes in each pathway.

Based on KEGG pathway analysis 2,503 (19.27%) unigenes were primarily classified with Enzyme Commission (EC) numbers, and further classified into six branches of Metabolism, Cellular Processes, Genetic Information Processing, Environmental Information Processing, Organismal Systems and Human diseases, and they might be further clustered into 314 KEGG pathways (Fig. 1). It was noteworthy that 910 (36.36%) unigenes were grouped into the metabolism, 411 (16.42%) unigenes were involved in Genetic Information Processing, 408 (16.30%) unigenes were involved in human diseases, 301 (12.03%) unigenes were involved in Organismal systems, 248 (9.91%) unigenes were involved in Cellular Processes, and 225 (8.99%) unigenes were involved in the Environmental Information Processing. In addition, the most predominant 90 pathway of *A. apis* were presented in Fig. 1. Inside them, the most important and representative pathways were protein processing in endoplasmic reticulum (49 unigenes) biosynthesis of amino acids (43 unigenes), spliceosome (40 unigenes), and RNA transport (39 unigenes).

### Transcription factor analysis

Putative transcription factor (TFs) genes are often classified to different sub-classification based on their DNA-binding domains. In the present data study, a total of 651 unigenes were annotated successfully, among these TF unigenes, particularly, functional annotation revealed that 356 transcriptional factor (TF) genes typically found in fungi (3.71% of the 9598 protein-coding genes in *A. apis* in this study). According to InterPro classification^18^, a total of 356 *A. apis* TF genes were clustered into 32 families. Among them four families were dominating: Zn2Cys6 Zn_cluster (209 genes; 58.7%), basic-leucine zipper (BZIP) TF (23 genes; 6.5%), zinc finger CCCH-type (21 genes; 5.9%), and Helix-loop-helix DNA-binding (13 genes; 3.7%) (Supplementary information, Fig. S3). Furthermore, six genes involved in more than one DNA-binding domains.

### Comparative transcriptome analysis

**Figure 2:**
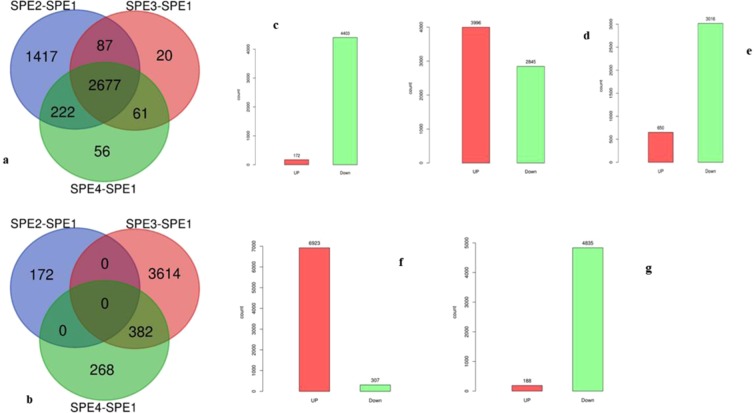
Differentially expressed genes (DEGs): (**a**) Venn-diagram of down regulated, (**b**) Venn-diagram of up-regulated genes, (**c**) DEGs between SPE2-SPE1, (**d**) DEGs between SPE3-SPE1, (**e**) DEGs between SPE4-SPE1, (**f**) DEGs between SPE3-SPE2, and (**g**) DEGs between SPE4-SPE3.

In the present study, comparative transcriptome analysis were carried out at the gene expression changes over absolute value of two-fold change (log2|FC > 1|). A total of 2,677 genes were commonly expressed lower in all three mutants compared to their original type of *A. apis*. However, 382 genes were commonly up-regulated both in SPE3 and SPE4 compared to the wild type (Fig. 2a,b). Overall, we detected 172, 3,996, and 650 up-regulated DEGs and 4,403, 2,845, and 3,016 down-regulated DEGs between each of the mutants and wild-type libraries (SPE2-SPE1, SPE3-SPE1, and SPE4-SPE1) (Fig. 2c–e), respectively. In addition, we detected 6,923, and 188 up-regulated DEGs and; 307 and 4,835 down-regulated DEGs between the SPE3- SPE2 libraries and the SPE4-SPE3 libraries, respectively (Fig. 2f,g, Supplementary information, Fig. S4). Top ten down- and up-regulated genes in each mutant compared to SPE1 are presented in Tables 3, 4, 5.

**Table 3:** The 10 most up- and down-regulated genes in *A. apis* genes (SPE2-SPE1).

Comparison	Gene ID	Type	Log2 (FC)	Description
SPE2-SPE1	Cluster-7978.0	Up	9.00	S9VMK0, Triosephosphate isomerase
SPE2-SPE1	Cluster-10688.0	Up	8.74	A0A167PS18, Glycerol 2-dehydrogenase
SPE2-SPE1	Cluster-10311.1	Up	8.61	A0A167T893,60 S ribosomal protein
SPE2-SPE1	Cluster-10513.0	Up	8.61	A0A167VQB3,60 S ribosomal protein L36
SPE2-SPE1	Cluster-10697.0	Up	8.01	A0A161ZCJ3, Uncharacterized protein
SPE2-SPE1	Cluster-10577.0	Up	7.91	A0A167SPY0, Acetyltransferase component of pyruvate dehydrogenase complex
SPE2-SPE1	Cluster-10542.0	Up	7.89	A0A167VG04, Putative redox protein
SPE2-SPE1	Cluster-10419.0	Up	7.78	A0A167YBY5, High mobility group protein
SPE2-SPE1	Cluster-10672.0	Up	7.65	A0A117NLB1, Uncharacterized protein
SPE2-SPE1	Cluster-10559.0	Up	7.50	A0A1V6NC47, Uncharacterized protein
SPE2-SPE1	Cluster-5822.1	Down	−11.19	A0A179HTK0, Transmembrane GTPase Fzo1
SPE2-SPE1	Cluster-6486.1	Down	−10.76	A0A179GBQ6, C2HC5 finger protein
SPE2-SPE1	Cluster-6028.0	Down	−10.49	A0A2C5YSX2, Uncharacterized protein
SPE2-SPE1	Cluster-7741.0	Down	−10.47	A0A179GJ91, Vacuolar sorting protein 1
SPE2-SPE1	Cluster-9111.0	Down	−10.33	A0A179GE34, Trehalase
SPE2-SPE1	Cluster-7335.0	Down	−10.30	A0A179GI75, Methylenetetrahydrofolate Reductase
SPE2-SPE1	Cluster-6407.0	Down	−10.28	A0A179HFX0, T-complex protein 1 subunit
SPE2-SPE1	Cluster-5298.0	Down	−10.21	A0A179GJM7, Las1-like domain-containing Protein, A0A179H6R8, Las1-like protein
SPE2-SPE1	Cluster-8155.0	Down	−10.21	A0A179GP36, Phospholipid:diacylglycerol Acyltransferase
SPE2-SPE1	Cluster-8518.0	Down	−10.19	A0A179HHE4, Conserved membrane protein

**Table 4:** The 10 most up- and down-regulated genes in *A. apis* genes (SPE3-SPE1).

Comparison	Gene ID	Type	Log2 (FC)	Description
SPE3-SPE1	Cluster-4091.0	Up	12.75	T0KQB0, AhpC/TSA family protein
SPE3-SPE1	Cluster-851.0	Up	11.17	
SPE3-SPE1	Cluster-4506.0	Up	10.44	A0A0F2MHU1, C6 zinc finger domain containing protein, R8BH37, Putative fungal specific transcription factor domain-containing protein
SPE3-SPE1	Cluster-10511.0	Up	10.43	R8BGV6, Putative integral membrane protein
SPE3-SPE1	Cluster-9946.0	Up	10.40	A0A1Q8RX65, Uncharacterized protein
SPE3-SPE1	Cluster-3385.0	Up	10.38	R8BMG1, Putative hsp20-like protein
SPE3-SPE1	Cluster-2451.0	Up	10.32	R8BTA4, Uncharacterized protein
SPE3-SPE1	Cluster-2925.0	Up	10.32	A0A167N866, Mannan endo-1,6-alpha-mannosidase
SPE3-SPE1	Cluster-3992.1	Up	10.31	R8BKG6, Cystathionine beta-synthase
SPE3-SPE1	Cluster-961.0	Up	10.24	R8BQU1, Putative bleomycin hydrolase protein
SPE3-SPE1	Cluster-8267.0	Down	−9.60	B8NCK6, Sensor histidine kinase/response regulator TcsB/Sln1, putative
SPE3-SPE1	Cluster-7392.0	Down	−9.49	I7ZXW6, Uncharacterized protein
SPE3-SPE1	Cluster-6719.0	Down	−9.15	I8IMJ4, Cell cycle control protein
SPE3-SPE1	Cluster-10038.0	Down	−9.10	Q5VDD7, OmtA A0A0D9MSH1, O-methyltransferase
SPE3-SPE1	Cluster-9472.0	Down	−8.96	A0A0D9MV28, Uncharacterized protein
SPE3-SPE1	Cluster-5863.0	Down	−8.87	A0A0D9MRT4, Uncharacterized protein
SPE3-SPE1	Cluster-5703.0	Down	−8.76	A0A0D9N9B4, Uncharacterized protein
SPE3-SPE1	Cluster-8855.0	Down	−8.74	B8ND83, Uncharacterized protein
SPE3-SPE1	Cluster-9835.0	Down	−8.72	A0A0D9MUG0, Uncharacterized protein
SPE3-SPE1	Cluster-9583.0	Down	−8.66	A0A0D9N055, Domain found in IF2BIF5

**Table 5:** The 10 most up- and down-regulated genes in *A. apis* genes (SPE4-SPE1).

Comparison	Gene ID	Type	Log2 (FC)	Description
SPE4-SPE1	Cluster-3360.0	Up	9.21	A0A0F7ZQT7, Uncharacterized protein
SPE4-SPE1	Cluster-3261.0	Up	8.92	A0A179HFS8, Uncharacterized protein
SPE4-SPE1	Cluster-4329.0	Up	8.76	A0A179HVY6, Phosphatidylinositol 3
SPE4-SPE1	Cluster-1247.0	Up	8.39	A0A179FQD2, Allantoate permease
SPE4-SPE1	Cluster-9153.1	Up	8.20	A0A179H215, SWIM zinc finger protein
SPE4-SPE1	Cluster-2784.0	Up	8.19	
SPE4-SPE1	Cluster-802.1	Up	8.13	
SPE4-SPE1	Cluster-1720.1	Up	8.03	
SPE4-SPE1	Cluster-1071.0	Up	7.82	
SPE4-SPE1	Cluster-1025.0	Up	7.64	A0A179GDN0, Proteinase aspergillopepsin II
SPE4-SPE1	Cluster-8390.0	Down	−11.89	B8NCX4, Fasciclin domain family protein
SPE4-SPE1	Cluster-9725.0	Down	−11.49	Q2U575, Uncharacterized protein
SPE4-SPE1	Cluster-10301.0	Down	−10.65	B8MYB6, Uncharacterized protein
SPE4-SPE1	Cluster-9556.0	Down	−10.61	
SPE4-SPE1	Cluster-7766.0	Down	−10.37	
SPE4-SPE1	Cluster-10318.0	Down	−10.32	B8NDW9, Cell cycle checkpoint protein
SPE4-SPE1	Cluster-10251.0	Down	−10.20	I8U1U4, Uncharacterized protein A0A1S9DAJ4, Uncharacterized protein
SPE4-SPE1	Cluster-6556.0	Down	−10.19	I8IPA8, Uncharacterized protein A0A1Z5T6S4, Uncharacterized protein
SPE4-SPE1	Cluster-10407.0	Down	−10.16	A0A0D9N8T6, Amino acid permease
SPE4-SPE1	Cluster-9672.0	Down	−10.15	B8NRN8, Uncharacterized protein B8NRN8, Uncharacterized protein

### Virulence and pathogenesis related genes

One hundred genes encoding hydrolytic enzymes were found to be down-regulated in the mutants compared to the wild-type, including three chitinases, 32 proteases, 39 esterases, 7 lipases, 17 amidases and 2 cellulases, degrading enzymes that have implicated to be involved in virulence through host invasion and escape process (Supplementary information, Table S1). Furthermore, genome annotation reveals that a number of genes encoding homologs with a well-known toxin were down-regulated in mutants such as 34 polyketide synthase dehydratase (PksA) genes, mycotoxin biosynthesis protein UstYa-like (cluster-7579) and Zeta toxin (cluster-9806), some of them involved in the aflatoxin biosynthesis pathway (cluster-6333.0, cluster-8356.2, cluster-6835.0, cluster-9644.0, cluster-6655.0, cluster-8356.0, cluster-8356.1). In addition, the transcriptome analysis shows that there are several genes down-regulated in mutants: five pathogen genes, six virulence genes, five effector genes, four genes involved in sporulation, five melanogenesis genes and six genes encoding secreted proteins (Supplementary information, Table S1).

Furthermore, 122 genes encoding hydrolytic enzymes were found to be up-regulated in the mutants compared to the wild-type, including 30 proteases, 43 esterases, 26 amidases, seven lipases, seven chitinases, five cutinases and two cellulases, two lysozyme genes, degrading enzymes that have implicated to be involved in virulence through host invasion and escape process. Moreover, genome annotation reveals that a number of genes encoding homologs with a well-known toxin were up-regulated in mutants such as 28 polyketide synthase dehydratase (PksA) genes, mycotoxin biosynthesis protein UstYa-like, killer toxin, toxin, and alpha/beta hydrolase fold-1. In addition, the transcriptome analysis shows that there are several genes up-regulated in mutants: five pathogen genes, five virulence genes, five effector genes, four genes involved in sporulation, one melanogenesis gene, two immune genes, four secreted proteins.

### Regulation and signaling

Twelve regulatory proteins were down-regulated in mutants compared to wild-type. Among these, 11 regulatory proteins (cluster-5069.0, cluster-7921.0, cluster-7977.1, cluster-7977.1, cluster-7977.1, cluster-6267.0, cluster-7043.0, cluster-8228.0, cluster-9644.0, cluster-9691.0 and cluster-8246.0), six regulatory proteins (cluster-6267.0, cluster-7043.0, cluster-8228.0, cluster-9644.0, cluster-9691.0 and cluster-9907.0) and seven regulatory proteins (cluster-6267.0, cluster-7043.0, cluster-8228.0, cluster-9644.0, cluster-9691.0, cluster-8246.0 and cluster-9907.0) were down-regulated in SPE2, SPE3 and SPE4, respectively. Overall, thirteen regulatory proteins were found to be up-regulated in mutants compared to wild-type. Of these, 3, 10, and 1 regulatory protein were up-regulated in SPE2, SPE3 and SPE4, respectively. Five genes encoding HMG-box were found to be down regulated in mutants compared to wild type. Of these, four genes (cluster-6670.0, cluster-7777.0, cluster-8445.0 and cluster-9704.0) were down-regulated commonly in all mutants and one gene (cluster-8772.0) was down regulated in SPE3 only. On the other hand, 1, 6, and 1 genes were up-regulated in SPE2, SPE3, and SPE4, respectively. A total of 117 TF genes were found to be down-regulated in mutants compared to wild-type. Among the total TF genes, 112, 59 and 63 genes were down-regulated in SPE2, SPE3 and SPE4, respectively. Furthermore, a total of 117 TF genes were found to be up-regulated in mutants compared to wild-type. Among these, 4, 108 and 24 TF genes were up-regulated in SPE2, SPE3 and SPE4, respectively.

### Morphogenesis and development

Three DEGs cluster-192 (Ascus development protein which serves as sugar and other transporter), cluster-8834.1(SH3 domain containing protein which involved in cell morphogenesis) in SPE2 and cluster-8127.1 (interferon-related developmental regulator) in all the three mutants were down-regulated compared to wild type. Furthermore, one DEG Cluster-10371 (Fork head domain which involves in the transcription factor of Fork head/HNF3 family) was down-regulated and one DEG cluster-186 (Hsp 70 family chaperone, Cell shape determining protein MreB which involves in cell morphogenesis) was up-regulated in SPE4 compared to wild type. Furthermore, 95 genes involved in DNA repairing processes were identified. Among these, 66 genes were differentially expressed compared to wild type. In particular, 38, 18 and 18 genes were found to be down-regulated in SPE2, SPE3 and SPE4, respectively. Besides, 28 and three genes were found to be up-regulated in SPE3 and SPE4, respectively.

### Membrane proteins and transport

A total of 212 genes encoding transmembrane transporter proteins were identified. Among these, 81genes were found to be down-regulated in mutants compared to wild type. In particular, 78, 43 and 47 genes were down-regulated in SPE2, SPE3 and SPE4, respectively (Supplementary information, Table S1). Furthermore, a total of 79 and 12 transmembrane transporter proteins were found to be up-regulated in SPE3 and SPE4, respectively, compared to wild type (Supplementary information, Table S1).

### Cell wall related and detoxification genes expression

Twelve genes encoding glucanases were found to be down-regulated in mutants compared to wild type. Of these, twelve, six and seven genes were down-regulated in SPE2, SPE3 and SPE4 mutants, respectively. On the other hands, twelve genes encoding glucanases were found to be up-regulated in mutants compared to wild type. Of these, twelve and two genes were found to be up-regulated in SPE3 and SPE4 mutants, respectively. Sixteen genes encoding glucan were found to be down-regulated in mutants compared to wild type. Of these, sixteen, eight and nine genes were found to be down-regulated in SPE2, SPE3 and SPE4 mutants, respectively. Furthermore, a total of 22 genes encoding glucan were found to be up-regulated in mutants compared to wild type. Among these, two, eighteen and four genes were up-regulated in SPE2, SPE3 and SPE4 mutants, respectively. A total of 19 genes encoding GPI anchored proteins were down-regulated in mutants compared to wild type. Among these, nineteen, eight and eight genes were found to be down-regulated in SPE2, SPE3 and SPE4 mutants, respectively. On the other hands, a total of 14 genes encoding GPI anchored proteins were up-regulated in mutants compared to wild type. Of these, eleven and three genes were up-regulated in SPE3 and SPE4, respectively. In this study, of the total 23 detoxification genes identified, ten genes were found to be down-regulated in mutants compared to wild type. Of these, ten, six, and seven genes were down-regulated in SPE2, SPE3 and SPE4, respectively (Supplementary information, Table S1). In addition, six detoxification genes were up-regulated in mutant SPE3 only.

### Oxidation-reduction processes

A total of 106 genes encoding a cytochrome P450, heme-containing monooxygenases were identified. Among these, 70 genes were differentially expressed compared to wild type. In particular, 38, 20 and 22 genes were down-regulated in SPE2, SPE3 and SPE4, respectively. Besides, 37 and nine genes were up-regulated in SPE3 and SPE4, respectively.

### Gene ontology enrichment

**Figure 3:**
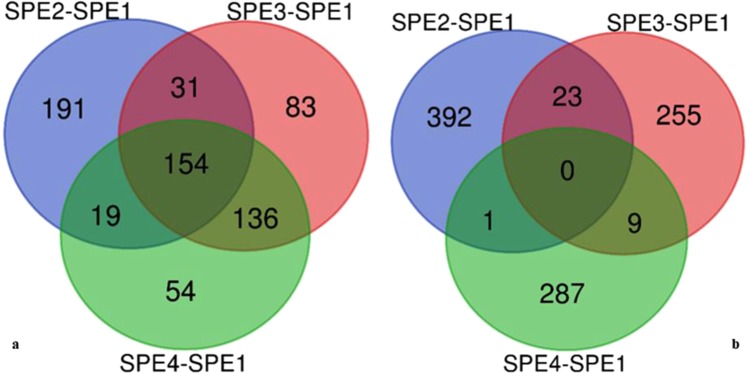
Venn diagram of DEGs for GO terms: (**a**) down regulated GO terms & (**b**) up-regulated GO terms.

The predominant functions of up- and down-regulated genes with the three GO categories (biological processes, cellular component, and molecular function) were assessed for each mutant in comparison to wild type. GO-term analyses showed that 395, 404, and 363 GO terms were down-regulated in SPE2, SPE3, and SPE4 mutants, respectively, in comparison to wild type (Fig. 3a). On the other hand, 416, 287, and 297 Go terms were up-regulated in SPE2, SPE3, and SPE4 mutants, respectively (Fig. 3b). The top 10 DEGs GO terms in each mutant compared to wild type are listed in supplementary information, Table S2–S7. The down-regulated gene ontology terms of molecular function category were concentrated in “tubulin binding (50 genes, representing 1.1% of all down-regulated genes” and “transferase activity, transferring alkyl...(48 genes, representing 1.1%” in SPE2, “ubiquitin-protein transferase activity (37 genes, representing 1.3%)” and “amino acid transmembrane transporter act...(23 genes, representing 0.8%)” in SPE3, and “structural molecular activity (109 genes, representing 3.6%” and “molecular function regulator (85 genes, representing 2.8%)” in SPE4 in comparison to wild type. The highest percentage of GO terms under cellular component category were “cell (3,106 genes, representing 70.5% of all down-regulated genes)” and “cell part (3,098 genes, representing 70.4%)” in SPE2, “cell part (2,004 genes, representing 70.4%)” and “cytoplasm (1,479 genes, representing 52%)” in SPE3, and “membrane-bounded organelle (1,416 genes, representing 46.9%)” and “organelle part (987 genes, representing 32.7%)” in SPE4 in comparison to wild type. In this study, GO terms were mainly categorized in to “biological process”, with wide distributions and extensive assignments than other categories. The most prevalent “biological processes” assignment were: “cellular macromolecule metabolic process (1,516 genes, representing 34.4% of all down-regulated genes)” and “cellular component organization or bioge... (953 genes, representing 21.6%)” in SPE2, “single-organism biosynthetic process (463 genes, representing 16.3%)” and “establishment of localization (462 genes, representing 16.2%)” in SPE3, and “single-organism biosynthetic process (487 genes, representing 16.1%)” and “establishment of localization (486 genes, representing 16.1%)” in SPE4, respectively, were highly enriched in comparison to wild type. This suggests that the biological processes of *A. apis* mutants were widely changed after REMI mutation.

### KEGG pathway

**Figure 4:**
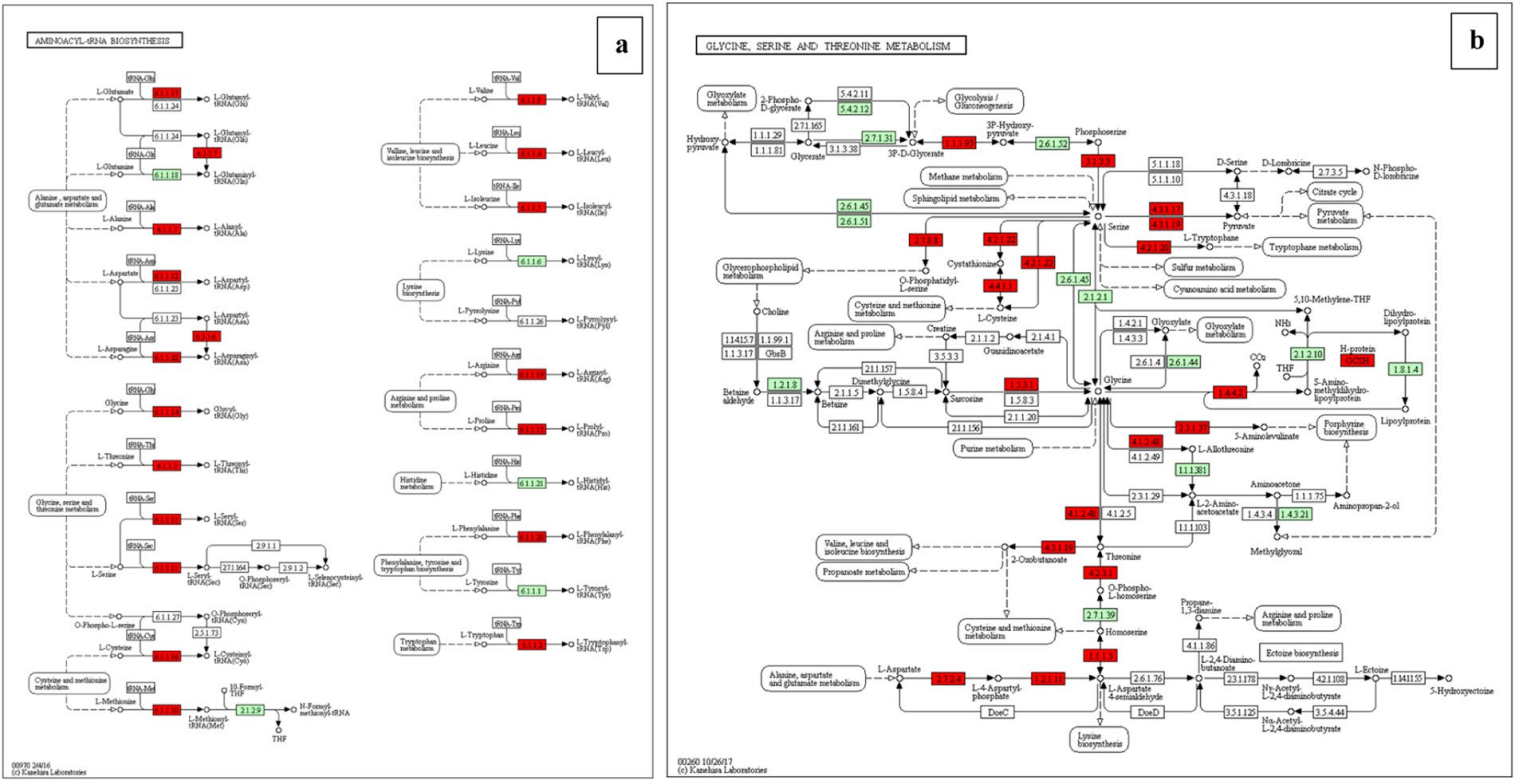
(**a**) enriched Glycine, serine and threonine metabolism and (**b**) enriched Aminoacyl-tRNA biosynthesis pathways with EC numbers found down-regulated in *A. apis* mutant SPE2 compared SPE1 shown in red color. EC numbers sown in green color are present in *Trichophyton verrucosum* KEGG database, but not identified in *A. apis* transcriptome^19^.

**Figure 5:**
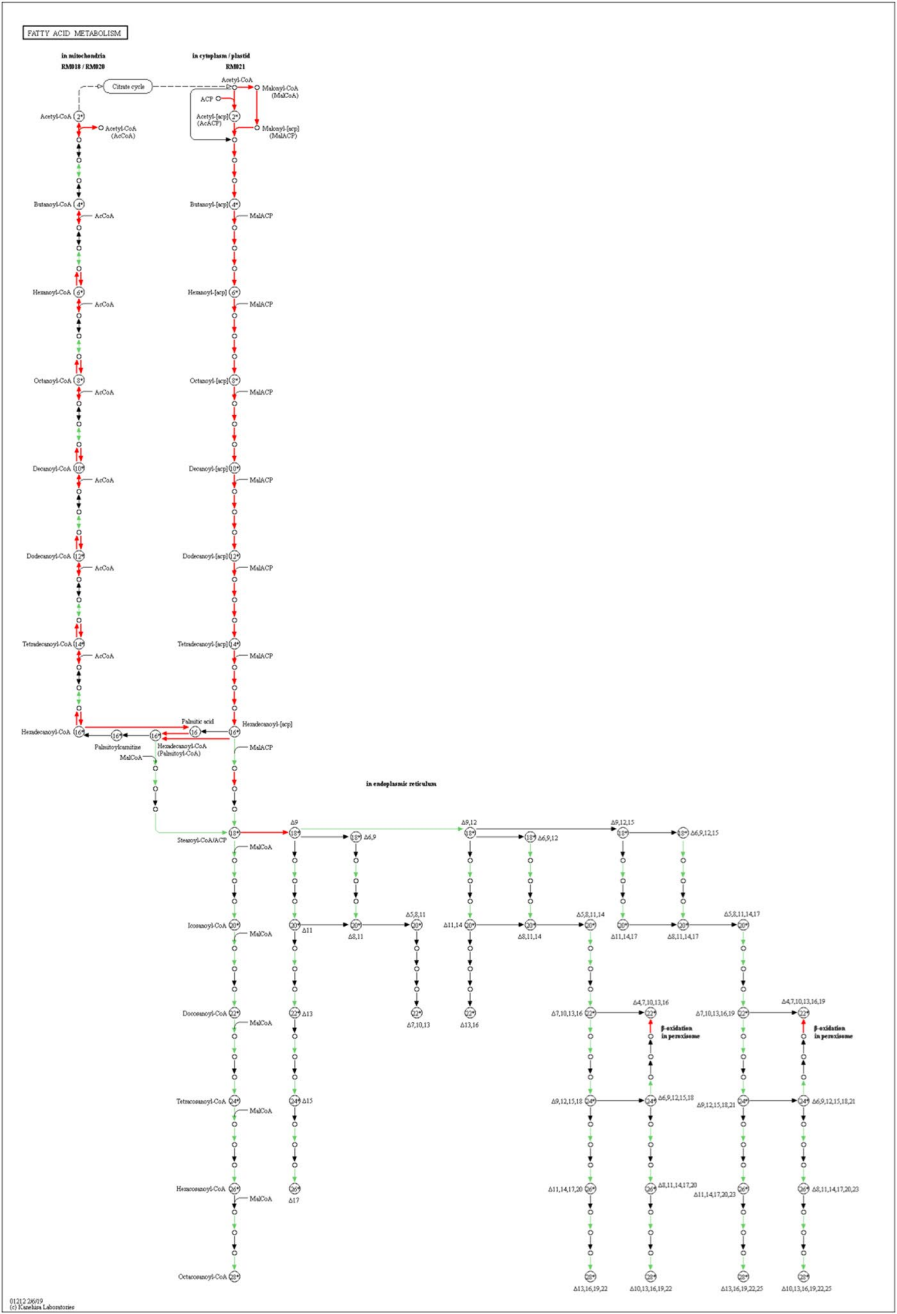
Enriched fatty acid metabolism pathway found down-regulated in *A. apis* mutant SPE4 compared to wild type (SPE1) shown in red color^19^.

**Figure 6:**
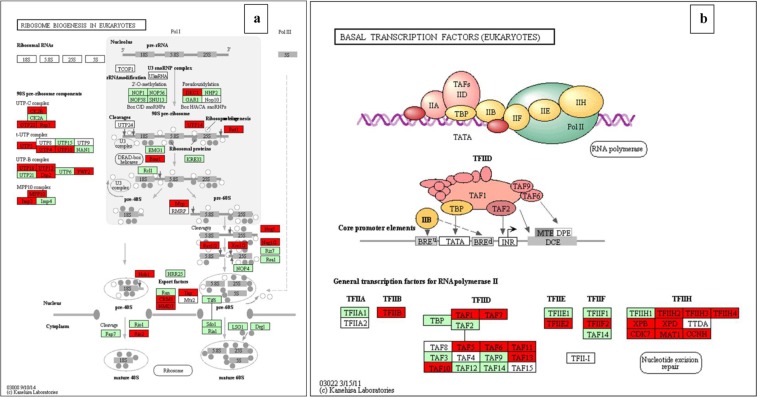
(**a**) enriched Ribosome biogenesis in eukaryotes pathway found up-regulated in *A. apis* mutant SPE3 compared to mutant SPE2 shown in red color and (**b**) enriched basal transcription factors pathway found down-regulated in *A. apis* mutant SPE4 compared to mutant SPE3 shown in red color^19^.

For better understanding of the biological function and correlation of DEGs (P value ≤ 0.05), we performed an enrichment analysis using KEGG pathway database, which assigned a total of 276 DEGs to 16 pathways (Table 6). The enriched pathway maps of DEGs are generated by mapping elementary datasets (genes, proteins and or small molecules) to KEGG pathway maps. All metabolism and genetic information processing pathways presented in this thesis are downloaded from open KEGG pathway database and analysis of DEGs is performed. In this study, Fisher testing and χ^2^ testing were used for pathway analysis to obtain the targeted significant pathway of the DEGs. Based on this analysis, five pathways were found to be involved in the up-regulated genes and 13 pathways were involved in the down-regulated genes. Most of these pathways are containing genes known to be associated with virulence. The up-regulated pathways including Oxidative phosphorylation (six genes), Ribosome biogenesis in eukaryotes (54 genes), Glycine, serine and threonine metabolism (33 genes), Terpenoid backbone biosynthesis (four genes), and Pentose phosphate pathway (two genes). The down-regulated pathways including Proteasome (28 genes), Glycine, serine and threonine metabolism (28 genes), Aminoacyl-tRNA biosynthesis (31 genes), Basal transcription factors(21 genes), SNARE interactions in vesicular transport (11 genes), Sulfur metabolism (10 genes), Fatty acid metabolism (17 genes), Fructose and mannose metabolism (16 genes), Spliceosome (12 genes), Oxidative phosphorylation (six genes), Valine, leucine and isoleucine biosynthesis (three genes), Pantothenate and CoA biosynthesis (three genes), and Butanoate metabolism (three genes). In the KEGG graph, the red highlighted boxes stand for the DEGs changed in each pathway. The pathway enrichment analysis map or KEGG graph is shown for the top nine most significant pathways^19^ (Fig. 4–6, Supplementary information, Fig. S5–S8).

**Table 6:** KEGG pathway enrichment of DEGs.

Comparison	Pathway	Pathway ID	DEGs-Size	Size	RF	P value
SPE2-SPE1-Dw	Proteasome	tve03050	28	33	1.55	0.014
SPE2-SPE1-Dw	Glycine, serine and threonine metabolism	tve00260	28	36	1.42	0.028
SPE2-SPE1-Dw	Aminoacyl-tRNA biosynthesis	tve00970	31	43	1.31	0.040
SPE2-SPE1-Up	Oxidative phosphorylation	tve00190	6	53	5.28	0.004
SPE3-SPE1-Dw	Proteasome	tve03050	28	33	2.39	0.000
SPE3-SPE1-Dw	SNARE interactions in vesicular transport	tve04130	10	13	2.17	0.045
SPE3-SPE1-Dw	Sulfur metabolism	tve00920	10	13	2.17	0.045
SPE4-SPE1-Dw	Proteasome	tve03050	28	33	2.26	0.001
SPE4-SPE1-Dw	SNARE interactions in vesicular transport	tve04130	11	13	2.25	0.035
SPE4-SPE1-Dw	Fatty acid metabolism	tve01212	17	25	1.81	0.038
SPE4-SPE1-Dw	Fructose and mannose metabolism	tve00051	16	24	1.77	0.048
SPE4-SPE1-Up	Terpenoid backbone biosynthesis	tve00900	4	21	2.35	0.032
SPE4-SPE1-Up	Glycine, serine and threonine metabolism	tve00260	5	36	1.72	0.049
SPE3-SPE2-Dw	Spliceosome	tve03040	12	72	4.36	0.000
SPE3-SPE2-Dw	Oxidative phosphorylation	tve00190	6	53	2.96	0.028
SPE3-SPE2-Dw	Pantothenate and CoA biosynthesis	tve00770	3	16	4.9	0.037
SPE3-SPE2-Dw	Valine, leucine and isoleucine biosynthesis	tve00290	3	16	4.9	0.037
SPE3-SPE2-Dw	Butanoate metabolism	tve00650	3	17	4.61	0.042
SPE3-SPE2-Up	Ribosome biogenesis in eukaryotes	tve03008	54	66	0.95	0.039
SPE3-SPE2-Up	Glycine, serine and threonine metabolism	tve00260	33	36	1.06	0.039
SPE4-SPE3-Dw	Basal transcription factors	tve03022	21	28	1.25	0.049
SPE4-SPE3-Up	Pentose phosphate pathway	tve00030	2	25	3.42	0.025

Up refers to up-regulated pathway and Dw to down-regulated pathway. DEGs-Size, number of differentially expressed genes that contribute to the enrichment of the term. Size, number of expressed genes associated with the term. RF, rich factor.

Furthermore, we have detected DEGs encoding signal transduction pathways in mutants: Mitogen-Activated protein kinase (MAPK) pathway which involved in fungal pathogenicity and stress responses in pathogenic fungi, and cAMP-dependent protein kinase pathway (PKA) which are important in the process of signal transduction. In this study, 29 genes were differentially expressed in MAPK pathway. Of these, 15 genes in SPE2, five genes in SPE3, and seven genes in SPE4 were found to be down-regulated compared to wild type. Furthermore, 14 genes in SPE3 and two genes in SPE4 were found to be up-regulated compared to wild type. Three genes were differentially expressed in PKA pathway, of these, two genes in SPE2 (cluster-7739.0 and cluster-8028.0), one gene in SPE3 (cluster-8028.0) and one gene in SPE4 (cluster-8028.0) were down-regulated, and two genes in SPE3 (cluster-3555.0 and cluster-7730.0) and one gene in SPE4 (cluster-7739.0) were up-regulated compared to wild type.

### Protein-protein interaction

**Figure 7:**
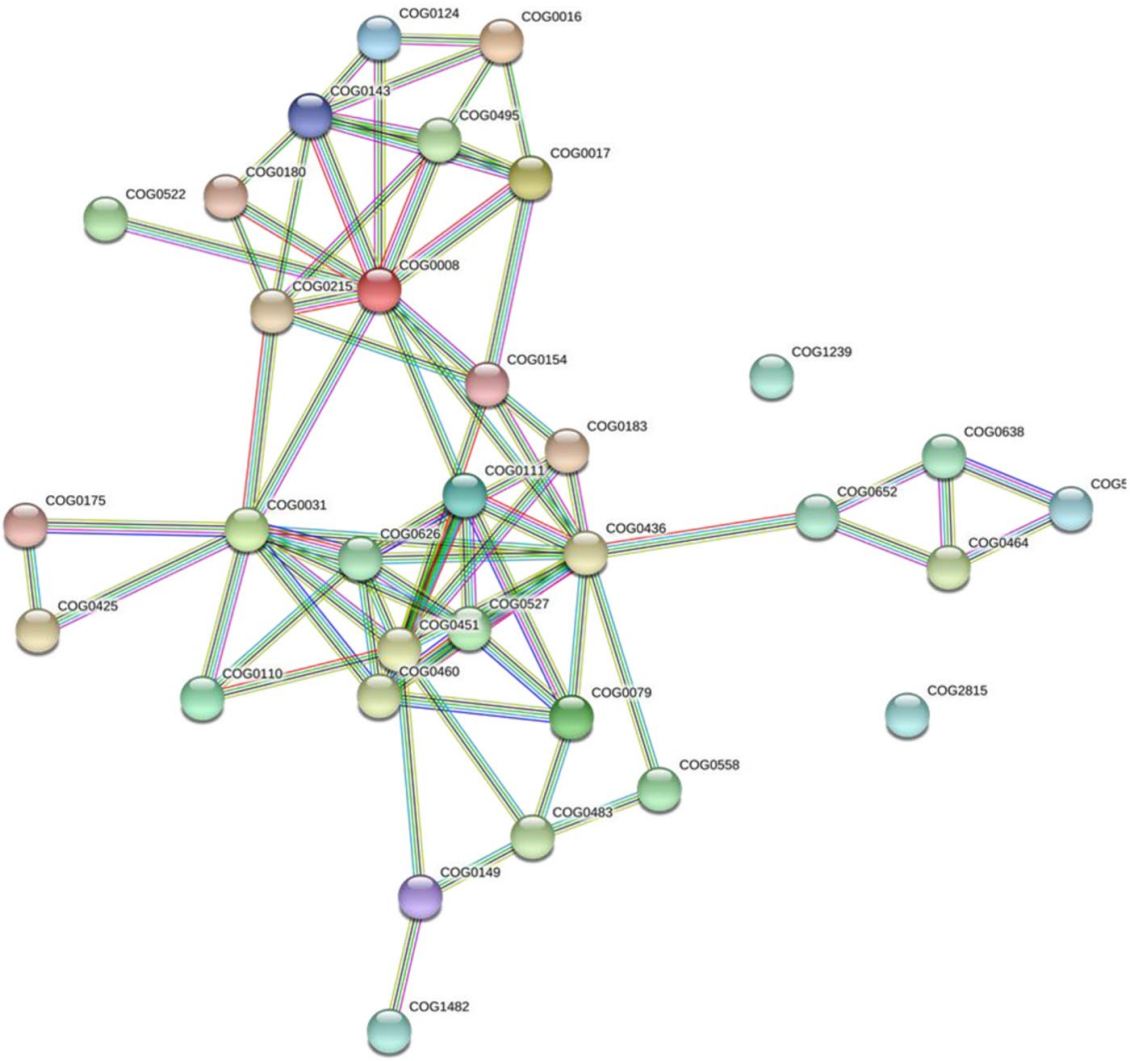
Global protein-protein interaction (PPI) networks for differentially expressed genes that altered by REMI mutation involved in nine pathways using STRING database, with COG functions. The nodes represent protein families and the lines represent the existence of the different types of evidence used in predicting the associations. A red line indicates the presence of fusion evidence, a green line shows neighborhood evidence, a blue line for co-occurrence evidence, a purple line indicates experimental evidence, a yellow line for text mining evidence, a light blue line stands for database evidence, and a black line for co-expression evidence.

The PPI networks analysis was conducted using the database (String database: https://string-db.org/cgi/input.pl, version 11.0) to protein families associated with metabolism and genetic information processing of *A. apis* mutants. The result revealed that, a total of 32 protein families that involved in nine pathways were detected in PPI analysis (Fig. 7, Supplementary information, Table S8). Of these, nine protein families were implicated in aminoacyl-tRNA biosynthesis: PF03950 (COG0008), PF00579 (COG0180), PF09334 (COG0143), PF03129 (COG0124), PF01409 (COG0016), PF00152 (COG0017), PF01425 (COG0154), PF01406 (COG0215), and PF00133 (COG0495). Eight protein families were involved in glycine, serine and threonine metabolism: PF00291 (COG0031), PF01053 (COG0626), PF03447 (COG0460), PF00696 (COG0527), PF00155 (COG0079), PF01221 (COG0436), PF02826 (COG0111), and PF01066 (COG0558). Four protein families in proteasome: PF00004 (COG0464), PF00225 (COG0638), PF01399 (COG5071), and PF13519 (COG1239). Four protein families involved in sulfur metabolism: PF01507 (COG0175), PF00581 (COG0425), PF00459 (COG0483), and PF01053 (COG0626). Three protein families were involved in fatty acid metabolism: PF00501 (COG0110), PF00108 (COG0183), and PF00107 (COG0451). Two protein families involved in fructose and mannose metabolism: PF00121 (COG0149) and PF01238 (COG1482). One protein family involved in basal transcription factor PF00069 (COG2815), one in ribosome biogenesis in eukaryotes PF01479 (COG0522), and one spliceosome protein family was involved in RNA recognition motif domain PF00076 (COG0652) that may play a crucial role in binding single-stranded RNAs.

### Quantitative Real Time PCR validation

**Figure 8:**
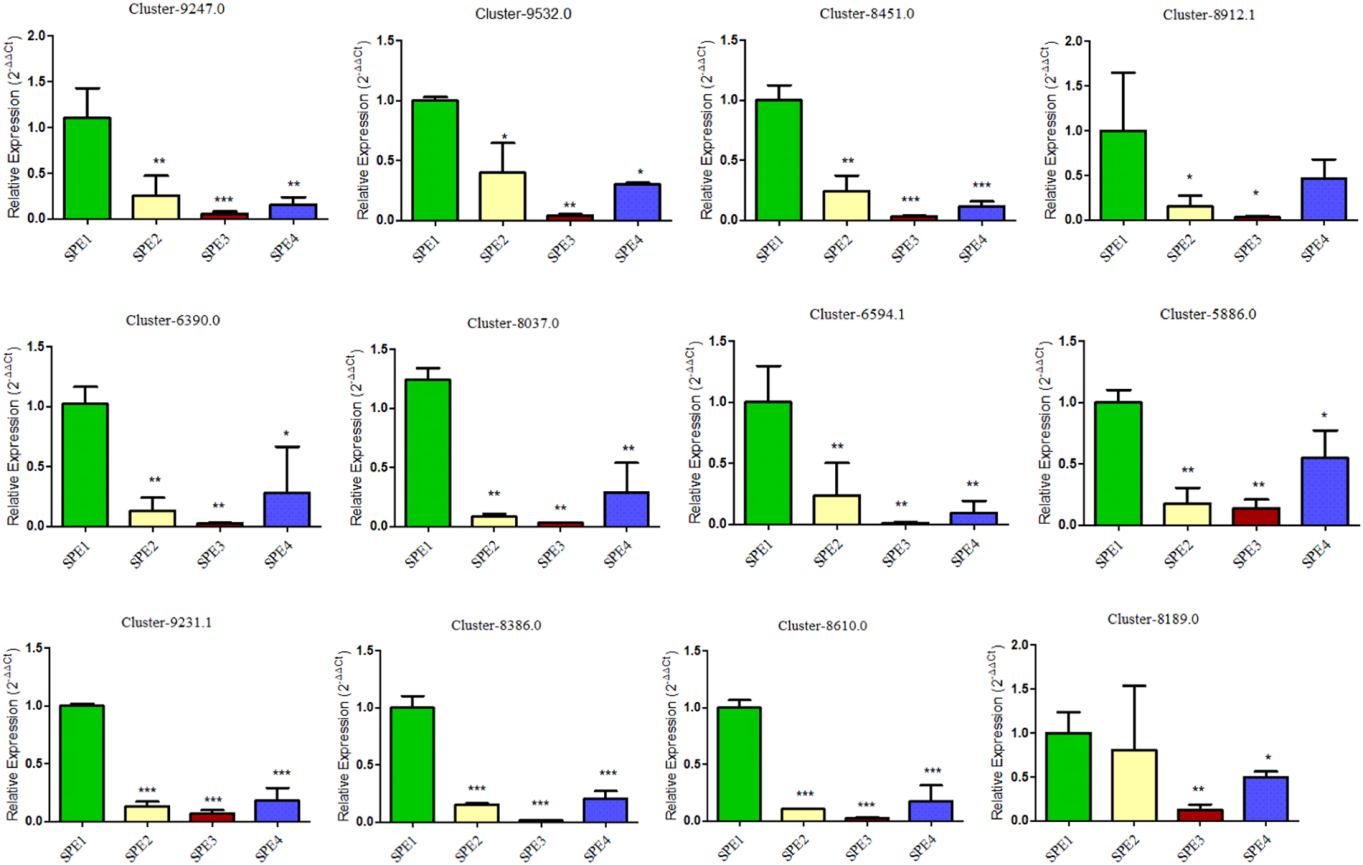
Relative expression of genes validated by qRT-PCR.

In this study, a total of 12 virulence related candidate genes found to be commonly down-regulated in SPE2, SPE3, and SPE4 were selected for validation to understand their expression level in in comparison to wild type strain. Quantitative Real Time PCR analysis reveals, all the 12 evaluated virulence related candidate genes were significantly down-regulated in all the three mutants compared to wild type (P < 0.05; Fig. 8).

## Discussion

The genomics of *A. apis* has been of great interest recently due to the increasing prevalence of chalkbrood disease. The *de novo* transcriptome assembly was performed based on a normalized composite sample comprising 12 samples to maximize the chance of gene detection. Normally, the normalization of treatment helps to reduce the redundancy of the cDNA library, improve the sequencing efficiency, and increase the discovery of rare genes accordingly. The present work seeks to publicize the availability of the annotated transcriptome of *A. apis* and assembled using the Trinity pipeline, and subsequently annotated using Trinotate. A reference-free functional annotation was achieved for *A. apis* using a homology search in the protein database. In total, 394,910,604 sequencing reads were produced and 12,989 unigenes were assembled. Among them, 9,598 (73.89%) unigenes were matching to UniProt Knowledgebase (UniProtKB), which is higher than previously reported 6,992 protein-coding genes in *A. apis*^20^. In this study annotation obtained predominantly from the UniProtKB which is a collection of accurate and consistent functional annotation of proteins^21^. The mean length of 758.9074 bp (with N50 length of 853 bp) are comparable to the results of *de novo* transcriptome assemblies in other entomopathogenic fungi species. This would help a great deal in identifying pathogenicity associated genes while infecting honeybee larvae.

Although several unigenes have not been annotated with functions, the present work provides about 9,598 annotated protein-coding genes similar to those of the average protein-coding genes in other entomopathogenic fungi^22^, to be directly further studied in *A. apis*. The major unannotated unigenes may be due to the current lack of a reference genome of *A. apis*, in addition, the data set might include a part of new *A. apis*-specific unigenes, since normalization of the fungal samples for *de novo* assembly should enhance gene detection and discovery. The complete transcriptome of the wild-type and the three mutants were constructed and annotated. Comparative analysis among orthologous transcripts revealed 172, 3,996 and 650 genes were up-regulated and 4,403, 2,845, and 3,016 genes were down- regulated between each of the mutants and wild-type libraries (SPE2-SPE1, SPE3-SPE1, and SPE4-SPE1), respectively. Furthermore, we detected 6,923 and 188 up-regulated DEGs and; 307 and 4,835 down-regulated DEGs between the SPE3-SPE2 libraries and the SPE4-SPE3 libraries, respectively (FC > 2, and FDR < 0.05).

The pathogenicity of entomopathogenic fungi is determined by the ability of its hydrolytic enzymes such as lipases, proteases, esterases, chitinases that degrade the insect’s integument^23^. The expression of a various hydrolytic enzymes and other factors endorse germination rate and growth performance of the fungus across the surface of the host, and the subsequent penetration of cuticular layers^24,25^. For instance, the expression of esterase gene (*Mest1*) is vital for virulence against caterpillars which allows to mobilize endogenous lipid reserves, promotes germination rate as well as infection structure formation^26^. Furthermore, pathogenic fungi use secreted molecules, termed as effectors proteins, that enable interaction of microbes with their hosts and influences the outcome of the interaction^27^. The present study revealed that 100 genes encoding hydrolytic enzymes were found to be down-regulated in the mutants compared to the wild-type (Supplementary information, Table S1) including 3 chitinase, 32 proteases, 39 esterases, 7 lipases, 17 amidases and 2 cellulases, degrading enzymes that have implicated to be involved in virulence through host invasion and escape process^20^.

Effective incidence of fungal pathogen is determined by its capability to thrive in stressful host niche colonization sites, to tolerate host immune system-induced stress and to resist antifungal drugs^28^. The overexpression of membrane transporter genes are reported to be involved in *Trichophyton rubrum*^29^ and in *Corynebacterium pseudotuberculosis* pathogenicity^30^. Multidrug resistance (MDR) transporters which belonging to the ATP-binding cassette (ABC) and the major facilitator superfamilies (MFS), known to play a key role in facilitating fungal resistance to pathogenesis-related stresses which likely to be linked to the general function of cellular detoxification ^28^. Our transcriptome analysis reveals that 81 genes involved in membrane transporter were down-regulated in mutants compared to wild type. In particular, 78, 43 and 47 membrane transporter genes were down-regulated in SPE2, SPE3 and SPE4, respectively.

Fungal transcription factors (TFs) play key roles in coordination of gene expression. The TF cohort describes the regulatory ability of an organism and the evolutionary history of TF families reflecting the history of the cognate regulatory mechanisms^31^. Successful fungal pathogenesis involves a well-orchestrated multiple cellular regulation and developmental processes in response to numerous stimuli from the host and the environment which is mainly regulated by TFs^32^. Therefore, TFs are recognized as critical proteins for fungal pathogenicity, as many of them are known to play vital roles in the transcriptional regulation of pathways implicated in virulence^33^. In this study, a total of 356 TF genes (3.7% of the 9,598 protein-coding genes) typically found in fungi are identified in *A. apis*, which is in line with previous research that reported TF genes represent 3–6% of the predicted genes in eukaryotic genomes^34^. Of the total 356 fungal type TF genes, 229 TF genes were found to be differentially expressed, 117 genes were down- and 117 genes up-regulated compared to wild type. Particularly, 112, 59, 63 genes down-regulated and 4, 108, 24 genes were up-regulated in SPE2, SPE3, and SPE4, respectively, compared to wild type. Furthermore, the trace element zinc contributes a key role for proper functioning of a large number of proteins, including various enzymes^35^.

Comparative analysis in this study revealed that numerous cell wall related genes were down-regulated in mutants compared to wild type (Supplementary information, Table S1). Innate immunity development in multicellular organisms is determined by the evolution of cell surface receptors that could detect molecules whose chemical pattern is conserved within numerous classes of foreign organisms but is absent in “self” molecules^36^. These “non-self” factors are named as Microbe/Pathogen-Associated Molecular Patterns (MAMPs/PAMPs). Members of large family of pattern recognition receptors (PRRs) recognized the presence of MAMPs or PAMPs in the host, which then activate signaling related pathways to persuade downstream defense responses^36^. The cell wall of fungi with a dynamic structures which composed of polysaccharides acts as a vital role to determine cell shape and shielding the host cells away from stresses. Fungal cell walls are responsible for the pathogenicity initiation process when infecting animals or plants^37^. This is because, fungal cell walls are complex and dynamic structures and crucial for its viability, morphogenesis and its pathogenesis. Normally, the turgor pressure of cell wall has been bioassayed to be between 0.2 and 10 MPa which is equivalent to 2 to 20 times atmospheric pressure, for instance, the melanized cell walls of the appressoria of some plant pathogens such as *Magnaporthe oryzae* can withhold the internal turgor pressure of up to 20 MPa which generate the force that empowers hyphae to exert mechanical force on the substrates they are penetrating^38^.

Fungal pathogens, in response to host defense, have developed sophisticated mechanisms to shield their chitin fibrils in hydrolysis and recognition by deaminases that convert chitin into chitosan and through masking of chitin with α-glucans^39^. Fungal cell walls which contain β-glucan polysaccharides are major structural component of fungal cell walls and known to stimulate immune responses when detected by the host immune cells which leaves the pathogen vulnerable^40^. For instance, β-glucan surface exposure during *Aspergillus fumigatus* germination period known to activate an *Atg5*-dependent autophagy pathway called LC3-associated phagocytosis (LAP), which promotes fungal killing^41^. However, some fungal pathogens have developed protective surface structures to evade such immune control mechanisms by reducing recognition of β-glucan by host cells either through masking of β -glucans beneath α-glucans or by enzymatic exclusion of any exposed β-glucan polysaccharides by the secreted glucanase Eng1^40^. In addition, melanin reported to inhibit activation of LAP by eliminating the *p22phox* subunit from the phagosome^41^. As a result, melanization in *A. fumigatus* confers bluish grey color to conidia and required for pathogenicity which is a renowned virulence factor in mammal models^42^. The inner walls of many fungal spores contain complex amorphous polymerized phenolic compounds termed as melanins, which also add protection-particularly from oxidants and some exoenzymes^38^. In this study, five genes involved in melanogenesis found down-regulated in mutants compared to their original wild type strain of *A. apis*.

Furthermore, fungal pathogens produce numerous secondary metabolites that support them as weapons in limited environmental niches to compete against other organisms, carry out antibiotic, immunosuppression, and some virulence factors or toxins of pathogenic fungi in the process of host and pathogenic fungal interactions^43^. Several fungal Cytochrome P450, heme-containing monooxygenases, are involved in ergosterol synthesis, virulence formation and differentiations, as well as several toxic secondary metabolites production. In the present study, it has been identified that 70 genes encoding Cytochrome P450 were down-regulated in mutants compared to their original wild type. The repairing ability of DNA in a cell is critically important to the integrity of its genome for the normal functionality of an organism. It has been reported that many of the genes that were primarily shown to influence life span of an organism have turned out to be involved in the process of regulation of DNA damage repair and protection^44^. In this study, however, several genes involved in DNA repair were down-regulated in mutants.

Pathway enrichment analysis in this study reveals that genes involved in proteasome pathway including 28 genes were down-regulated in all the three mutants in comparison to wild type. Proteasomes are important to degrade unwanted or damaged proteins by proteolysis that breaks down peptide bonds. Protein biosynthesis and degradation retain a dynamic balance to properly sustain normal cell metabolism in an organism^45^. Consequently, all intracellular proteins and several extracellular proteins are continually being hydrolyzed to their constituent amino acids and replaced by the new synthesis^46^. Several studies reported that F-box proteins are vital in fungal pathogenicity^47^.

Aminoacyl-tRNAs biosynthesis pathway including 31 genes found to be down-regulated in SPE2 only compared to wild type. Aminoacyl-tRNAs are important substrates for translation and vital to determine how the genetic code to be interpreted as amino acids, hence, the function of aminoacyl-tRNA synthesis is to precisely match amino acids with tRNAs which containing the corresponding anticodon^48^. In addition to their translations functions, aminoacyl-tRNAs synthetases are implicated in various noncanonical functions such as gene transcription, mRNA translation, inflammation and immune response^49^. Glycine, serine and threonine metabolism is reported to be involved in bacterial pathogenesis^50^. In the present study, this pathway found to be down-regulated in SPE2 (28 genes) and up-regulated in SPE4 (5 genes) compared to wild type.

Fungal pathogens have to assimilate the available nutrients within host niches to infect them. To attain this, fungi regulate specific nutrient uptaking mechanisms, modulating their metabolism, displaying an impressive degree of metabolic flexibility. The metabolic flexibility that enhances the fitness of the fungus, is often as important for pathogenicity as virulence factors^51^. It has been reported that the breakdown of fatty acids is vital in the metabolism, development and pathogenicity of many fungi^52^. In this study, pathway enrichment analysis revealed that fatty acid metabolism including 17 genes was found to be down-regulated in SPE4 compared to wild type. Furthermore, mutation altered the expression of genes involved in primary metabolic processes in fructose and mannose metabolism including 16 genes were down-regulated in SPE4 compared to the wild type. It has been reported that sulfur metabolism involved in numerous metabolisms through S-adenosylmethionine (SAM), as a source of methyl groups, methylene groups, ribosyl groups, amino groups, aminopropyl groups and 50-deoxyadenosyl radicals^53^. Furthermore, sulfur metabolism plays an important role in the response to cadmium stress by the intermediary of glutathione^54^. Our study shows that ten genes involved in sulfur metabolism pathway were down-regulated in SPE3 compared to wild type.

A total of 10 and 11 genes involved in SNARE interactions in vesicular transport pathway were down-regulated in SPE3 and SPE4, respectively, compared to wild type. Membrane fusion is known to be driven by a cooperative action of SNARE proteins, which is a vital process in all living organisms that contributes to varieties of biological processes, for instance, cell fertilization and intracellular transport. Particularly, the numerous membrane-enclosed compartments in eukaryotic cells need to exchange their contents and communicate across membranes properly. Efficient and manageable fusion of biological membranes comprise the central components of the eukaryotic fusion machinery that are responsible for fusion of synaptic vesicles with the plasma membrane^55^.

It has been reported that successful fungal pathogenesis involves a well-orchestrated multiple cellular regulation and developmental processes in response to various stimuli from the host and the environment which is mainly regulated by TFs^32^. Therefore, TFs are recognized as important proteins for fungal pathogenicity, as many of them are known to play vital roles in the transcriptional regulation of pathways implicated in virulence^33^. Interestingly, in this study, KEGG enrichment analysis revealed that 21 genes involved in basal transcription factors found to be down-regulated in mutant SPE4 compared to mutant SPE3. In addition, 54 genes involved in ribosome biogenesis in eukaryotes found to be down-regulated in mutant SPE2 compared to mutant SPE3. Inhibition of ribosome biogenesis activity found to be used as effective action to control human pathogenic fungi^56^. Moreover, the gene encoding nucleolar Protein CgrA that functions in ribosome synthesis disrupted in *Aspergillus fumigatus* caused a delay in growth and found to be less virulent in immunosuppressed mice than wild type^57^.

In the present study, the lower DEG fold-change cutoff (log2|FC > 1| and integral analysis based on node centrality statistics were important for identifying candidate proteins which probably have remarkable roles in fungal pathogenesis. Moreover, in the present study, PPI network analysis revealed a total of 32 protein families were involved in metabolism and genetic information processing (basal transcription factors, aminoacyl-tRNA biosynthesis, spliceosome, ribosome biogenesis in eukaryotes, and proteasome) pathways. These proteins are encoded by a total of 66 genes enriched in altered pathways, thus these genes are predicted to be involved in *A. apis* pathogenicity from PPI network analysis (Table 6).

The present study, for the first time, compared the expression of pathogenic genes between the original wild-type strain and its REMI constructed mutants of *A. apis* with lower pathogenicity than the wild-type using transcriptomic methods. However, comparison of genes involved in pathogenicity in gene expression perspective and basic understanding of the mechanism of pathogenicity is a preliminary work. Therefore, in depth analysis of the pathogenic genes function and their interaction to the host organism should be studied further by gene editing technology and bioassay to the level of honeybee colony.

## Methods

### Samples

The wild-type (hereafter, SPE1) and the three mutants: mutant-1(hereafter, SPE2), mutant-4 (hereafter, SPE3) and mutant-7 (hereafter, SPE4) of *A. apis* constructed by Restricted Enzyme-Mediated Integration (REMI) technique were obtained from our previous study^7^, and the insertion of *hph* gene and its successful integration into host chromosomes was confirmed by PCR, Clustal W sequence alignment, and Southern blot analyses. In addition, *in vitro* bioassay confirmed mutants had notable differences in pathogenicity among themselves and with the wild-type strain. Accordingly, samples were selected based on their virulence level (SPE2-less pathogenic, SPE3- pathogenic, and SPE4- nonpathogenic) for comparative transcriptome analysis. confirmed insertion of *hph* gene and successful integration into host chromosomes. Following the standard techniques^1^, the wild-type and mutants of this fungus were grown for six days at 28 °C on Potato Dextrose Agar (PDA) plates supplemented with 1% yeast extract. Furthermore, plate cultures were provided 50 µg/mL hygromycin B as an antibiotic to inhibit the growth of bacteria. In addition, single layer growth medium plates were used to enhance sexual reproduction and sporulation of the fungal pathogen.

### RNA extraction

Total RNA was extracted from a total of 12 samples (3 replicates per treatment) made up by 100 mg of fungal hyphae and spore combined using TRIzol extraction reagent (Invitrogen Life Technologies, USA) according to the manufacturer’s protocol. Then, fungal DNA was removed from extractions with DNAseI followed by the removal of rRNA using Ambion’s Poly (A) Purist kit. The integrity of the RNA was detected by agarose gel electrophoresis and the concentration was determined using Nano Drop 2000 spectrophotometer (Thermo Scientific, Wilmington, DE, USA). The RNA integrity was further evaluated using an Agilent Technology 2100 Bioanalyzer (Agilent Technologies, USA).

### Library construction and sequencing

High-throughput sequencing using the Illumina sequencer (version 1.9) requires the construction of a sequence library to match it. The main construction process of the mRNA-seq library we used in this study was as follows: after the total RNA sample was qualified, 5 g of total RNA was taken to carry out the subsequent database building experiment. Enrichment of eukaryotic mRNA by magnetic beads with Oligo (dT) (for prokaryotes, enrichment of mRNA by removal of rRNA by a kit), and then added fragmentation. Buffer breaks the resulting mRNA into a short clip. Using mRNA as template, one-strand cDNA was synthesized with six-base random primers, and then buffer, dNTPs and DNA were added. Polymerase I synthesized the second chain cDNA. Eluted purified double-stranded cDNA and then carried out terminal repair, base A was added, sequencing connector treatment was added, cDNA 5′ end connection UID connector, using magnetic beads to recover the target size fragments and PCR amplification. The constructed library was tested by agarose electrophoresis. Take advantage of Qubit 2.0 library was quantified to determine whether the library concentration was suitable for computer use. After the library was qualified, the library was sequenced on the Illumina sequencer according to the demand of effective concentration and target data.

### Transcriptome *de novo* assembly

Using short sequence assembly software Trinity to assemble *de novo* assembly, the transcription sequence was obtained by “cocoon-pupa-butterfly” three steps. The longest transcript is usually selected as unigene for subsequent annotation, quantification, and differential expression analysis. However, studies have shown that unigene is inappropriate as a surrogate for genes. For the longest transcribed copy that is spliced will mask the reference sequence meaning of the true shorter transcribed copy (isoform of the gene). Here, we use the Corset software [1,2] recommended by Trinity to filter and cluster the spliced transcripts to get closer to the real “gene”, breaking through the traditional concept of “unigene”. Trinity software v.2.1.1 (https://github.com/trinityrnaseq/ trinityrnaseq/wiki) was used for *de novo* assembly of the transcriptomes using sequencing data from the total of 12 libraries (Table 1). The default assembly parameters of Trinity were used, with the addition of the “–jaccard clip” function, because a high gene density with overlapping of UnTranslated Region (UTR) was expected^58^. Transcriptome completeness was assessed using BUSCO v3.0.2 (Benchmarking Universal Single-Copy Orthologs; python /home/nan/anaconda3/bin/run_BUSCO.py -i Trinity.fasta -o trinity -l /home/nan/database/busco/fungi_odb9/ -m transcriptome -c 16). In addition, Principal Component Analysis (PCA) of samples was examined to look at replicate clustering and separation of mutant samples from the wild-type.

### Functional annotation

The annotation of putative genes obtained from each assembly was performed using Basic Local Alignment Search Tool X (BLASTx) with an expectation value of 10^−5^ to search the following protein databases: Non-Redundant (NR) protein database of National Center for Biotechnology Information (NCBI), UniProt, and Kyoto Encyclopedia of Genes and Genomes (KEGG). Subsequently, protein information and their respective functional annotations were retrieved for genes with the highest sequence similarity with *A. apis* clusters (unigenes). The gene function terms were obtained from Gene Ontology (GO) annotation (http://www.geneontology.org) database using Blast2GO (http://www.blast2go.com/b2ghome). Functional classification of genes was conducted using COG (Clusters of Orthologous Groups of proteins, http://www.ncbi.nlm.nih.gov/COG/), and pathway annotation was carried out using KEGG (http://www.genome.jp/kegg/).

### Identification of transcription factors

Transcription factors known as sequence specific DNA binding factors, are the proteins that bind to a specific DNA sequences, play a vital role in controlling gene transcription activity^59^. Identification of transcription factors as a byproduct in the transcriptome is cost-effective and reliable. Therefore, the assembled transcriptome of *A. apis* was also analyzed for the identification of transcription factors. The A. apis transcripts were searched against all the transcription factor protein sequences available at Fungal Transcription Factor Database (http://ftfd.snu.ac.kr/).

### Comparative transcriptome analysis

Differentially expressed genes (DEGs) were identified based on the negative binomial distribution with the edgeR package^60^. False discovery rate (FDR) values of the genes was primarily calculated by using edgeR, and mapped reads numbers of genes were used for the analysis. The genes with FDR < 0.01 were considered as candidates. In addition, fragments per kilobase of gene per million mapped reads (FPKM) of these candidates were generated by using RSEM^61^. Finally, the fold change of FPKM was computed, and genes with the over the absolute value of two-fold change (log2|FC > 1|) were characterized as DEGs. Functional enrichment analyses were then performed on identified DEGs by using GO stats^16^.

### Gene ontology categories enrichment analysis

To determine the functional category, the DEGs were mapped to the GO database (www.geneontology.org) by GOEAST tool for each mutant in comparison to wild type. The biological process, cellular components and molecular functions that were particularly over- or under-represented in DEGs were extracted and visualized through GOEAST (omicslab.genetics.ac.cn/GOEAST/), which generally referred to as GO analysis. The Q-value is the corrected P-value with threshold < *0.05*.

### KEGG pathway enrichment

For KEGG pathway analysis, hypergeometric test function was used (p < 0.001)^19^. In order to elucidate the significant pathways based on KEGG database, pathway analysis was performed for DEGs detected in the first step. In this study, Fisher’s exact testing and χ^2^ testing were used for pathway analysis to obtain the targeted significant pathway of the DEGs that are altered due to REMI mutation. The rich factor is calculated as the ratio of the numbers of DEGs enriched in a particular pathway, to the total number of annotated genes in the same pathway of interest.

### Protein-protein interaction network construction

In order to interpret the molecular mechanisms of the main cellular activities in *A. apis* mutants constructed with REMI, the online Search Tool for the Retrieval of Interacting Genes (STRING) (http://string-db.org/) was used for constructing a protein–protein interaction (PPI) network of the DEGs enriched in KEGG pathways. Clusters of Orthologous Groups (COGs) of proteins functions were used in constructing the protein-protein interaction networks. Only an interaction networks with a high confidence (0.700) were retained. Furthermore, the eukaryotic orthologous groups (KOGs) were considered prime selection of a single protein spot.

### Validation of transcriptome data with quantitative real time PCR

To validate results of our *de novo* RNA-seq, 12 commonly down-regulated genes in mutants SPE2, SPE3 and SPE4 were selected to qRT-PCR analysis. The genes are involved in KEGG pathways: five, four, and three genes were involved in proteasome, SNARE interactions in vesicular transport, and fatty acid metabolism, respectively (Table 7). We compared the expression level of the selected candidate virulence related genes in mutants against to wild type *A. apis* strain in triplicates. The *A. apis actin* gene was used as a reference gene for normalizing qRT-PCR validation since it has been reported to be among the most stable genes in *A. apis*^62^. Gene specific primers were used to generate specific PCR fragments in *A. apis* pathogenicity associated genes (Table 8). Here, all primer pairs were designed using PrimerExpress 3 Software (Life Technologies) following the standard procedure. The Relative quantification of a gene is defined as the change in expression of the target gene relative to the reference groups such as untreated control and/or a sample at time zero in a time-course study^63^. The relative expression level of each gene was calculated by the formula (2^−∆∆CT^)^63^. The mean differences between original and mutant strains in terms of relative gene expression were compared using one-way ANOVA and student’s t-test.

**Table 7:** Genes used for qRT-PCR analyses.

Gene ID	Pathway	SPE2-SPE1	SPE3-SPE1	SPE4-SPE1
Cluster-9247.0	Proteasome	−6.5	−6.5	−6.5
Cluster-9532.0	Proteasome	−9.1	−9.1	−9.1
Cluster-8451.0	Proteasome	−9.2	−9.2	−9.2
Cluster-8912.1	Proteasome	−6.3	−6.3	−6.3
Cluster-6390.0	Proteasome	−7.4	−7.4	−7.4
Cluster-8037.0	SNARE interactions	−8.4	−8.4	−8.4
Cluster-6594.1	SNARE interactions	−6.3	−6.3	−6.3
Cluster-5886.0	SNARE interactions	−8.2	−8.2	−8.2
Cluster-9231.1	SNARE interactions	−5.3	−5.3	−5.3
Cluster-8386.0	Fatty acid metabolism	−6.1	−6.1	−6.1
Cluster-8610.0	Fatty acid metabolism	−6.1	−6.1	−6.1
Cluster-8189.0	Fatty acid metabolism	−7.1	−7.1	−7.1

**Table 8:** Primers used to see the expression of genes in qRT-PCR analysis.

Target gene	Forward primer (5′ to 3′)	Reverse primer (5′ to 3′)	Product size (bp)
Cluster-9247.0	GATCGACAACCCTCTTCCAA	TAAAACTGCACCGTGTCTCG	130
Cluster-9532.0	TGAGGGCTGCTTTCTTCAAT	CAGTGGCAGCTTGTTGTTGT	114
Cluster-8451.0	CTCTGCCGGTCTAGTTCCAG	CAGGGATAGGGCCCTTGTAT	95
Cluster-8912.1	GGCATCTCGAAAGTCACCTC	TTTGGAAAGCATCCAACTCC	101
Cluster-6390.0	TTCCATTGGTGGATCTGGTT	TCCCCGAACAAAGTTAATGC	100
Cluster-8037.0	AACGCAAGTTCCTATCCACCT	AGCGGAGCTCATTGTTGAAT	93
Cluster-6594.1	TTCCAAGGTCCTCGATGAGT	CCTGGTACGCCGAGATGTAT	104
Cluster-5886.0	TACAATTGCAGAGGCAGACG	TAGCAATGCCCAGTTCCTTC	93
Cluster-9231.1	GTCTCAGTTCAGCGGACACA	TGACTTTGAAGGAGGGTGCT	127
Cluster-8386.0	TGGTTTCCCCGAGACTACTG	ATCATGCCGGACTTGATAGC	102
Cluster-8610.0	ACCTGATCCTTGCCATTCTG	ATCGGGATTCGAGTTCTGTG	111
Cluster-8189.0	GAGGAGGCGAGTCTGAAATG	GACGTATTGCTGCGAGTTGA	99
*Ascosphaera apis Actin*	CATGATTGGTATGGGTCAG	CGTTGAAGGTCTCGAAGAC	*Actin*

## Supplementary information

Supplementary Figures and Tables.

## Data Availability

Data generated in this study have been submitted to the NCBI/GenBank database at Bioproject ID PRJNA541453. All raw sequence data have been deposited in the Sequence Read Archive (https://dataview.ncbi.nlm.nih.gov/?search = SUB5583364) under the accession number SRR9021798–9021809.
