# Supplementary material for: Transcriptome profiling reveals insertional mutagenesis suppressed the expression of candidate pathogenicity genes in honeybee fungal pathogen, Ascosphaera apis
Source: Sci Rep. 2020 May 5;10:7532. doi: 10.1038/s41598-020-64022-3 (PMC7200787; doi:10.1038/s41598-020-64022-3)
Supplement: Supplementary file 1 — Supplementary Figures and Tables. [file 41598_2020_64022_MOESM1_ESM.docx]

**Transcriptome profiling reveals insertional mutagenesis suppressed the expression of candidate pathogenicity genes in honeybee fungal pathogen, *Ascosphaera apis***

Awraris Getachew^1,2+^, Tessema Aynalem Abejew^1,2+^, Jiangli Wu^1^, Jin Xu^1^, Huimin Yu^1^, Jing Tan^1^, Pengjie Wu^1^, Yangyang Tu^1^, Weipeng Kang^1^, Zheng Wang^1^, Shufa Xu^1^*

^1^Key Laboratory of Pollinating Insect Biology, Ministry of Agriculture; Institute of Apicultural Research, Chinese Academy of Agricultural Sciences, 100093, Beijing, China

^2^College of Agriculture and Environmental Sciences, Bahir Dar University, Bahir Dar, Ethiopia

*corresponding author: [xushufa@caas.cn](mailto:xushufa@caas.cn)

^+^these authors contributed equally to this work and listed as first authors

**Supplementary information legends**

Supplementary information, Fig. S1. Principal Component Analysis (PCA) of mutants and wild-type. PCA analysis showed the mutant samples clearly separated from wild-type, indicating a visible variation among the different samples.

Supplementary information, Fig. S2. KOG functional classification of the annotated genes. A total of 230 annotated unigenes were assigned to 23 classification categories. The KOG categories are shown on the x-axis and gene proportions are plotted on the y-axis.

Supplementary information, Fig. S3. Summary of fungal specific transcription factor unigenes of *A. apis.* The vertical axis shows the number of unigenes and the horizontal axis represents TF families to annotated to *A. apis*.

Supplementary information, Fig. S4. Volcano plot showing the distribution of differentially expressed genes: a/ SPE2-SPE1, b/ SPE3-SPE1, and c/ SPE4-SPE1. Each point represents a single gene. The red dots are representing the up-regulated differentially expressed genes between mutant and wild type strains, green dots are representing the down-regulated differentially expressed genes, and black dots are representing non-differentially expressed genes.

Supplementary information, Fig. S5. Enriched Fructose and mannose metabolism pathway with EC numbers found down-regulated in *A. apis* mutant SPE4 compared to wild type (SPE1) shown in red color. EC numbers sown in green color are present in *Trichophyton verrucosum* KEGG database, but not identified in *A. apis* transcriptome^1^.

Supplementary information, Fig. S6. Enriched sulfur metabolism pathway with EC numbers found down-regulated in A. apis mutant SPE3 compared to wild type (SPE1) shown in red color. EC numbers sown in green color are present in *Trichophyton verrucosum* KEGG database, but not identified in *A. apis* transcriptome^1^.

Supplementary information, Fig. S7. Enriched proteasome pathway found commonly down-regulated in all the three *A. apis* mutants (SPE2, SPE3 and SPE4) compared to wild type (SPE1) shown in red color^1^.

Supplementary information, Fig. S8. Enriched SNARE interactions in vesicular transport pathway found down-regulated in two *A. apis* mutants (SPE3 and SPE4) in comparison to wild type (SPE1) shown in red color^1^.

Supplementary information, Table S1. Down-regulated genes associated with pathogenicity of *A. apis*

Supplementary information, Table S2. Top 10 GO enriched functions of down-regulated genes in SPE2-SPE1separated in to 3 GO term categories. DEGs-Size, number of differentially expressed genes that contribute to the enrichment of the term. Size, number of expressed genes associated with the term. FDR, false discovery rate., RF, rich factor.

Supplementary information, Table S3. Top 10 GO enriched functions of down-regulated genes in SPE3-SPE1separated in to 3 GO term categories. DEGs-Size, number of differentially expressed genes that contribute to the enrichment of the term. Size, number of expressed genes associated with the term. FDR, false discovery rate., RF, rich factor.

Supplementary information, Table S4. Top 10 GO enriched functions of down-regulated genes in SPE4-SPE1separated in to 3 GO term categories. DEGs-Size, number of differentially expressed genes that contribute to the enrichment of the term. Size, number of expressed genes associated with the term. FDR, false discovery rate., RF, rich factor.

Supplementary information, Table S5. Top 10 GO enriched functions of up-regulated genes in SPE2-SPE1separated in to 3 GO term categories. DEGs-Size, number of differentially expressed genes that contribute to the enrichment of the term. Size, number of expressed genes associated with the term. FDR, false discovery rate., RF, rich factor.

Supplementary information, Table S6. Top 10 GO enriched functions of up-regulated genes in SPE3-SPE1separated in to 3 GO term categories. DEGs-Size, number of differentially expressed genes that contribute to the enrichment of the term. Size, number of expressed genes associated with the term. FDR, false discovery rate., RF, rich factor.

Supplementary information, Table S7. Top 10 GO enriched functions of up-regulated genes in SPE4-SPE1separated in to 3 GO term categories. DEGs-Size, number of differentially expressed genes that contribute to the enrichment of the term. Size, number of expressed genes associated with the term. FDR, false discovery rate., RF, rich factor.

Supplementary information, Table S8. Protein families detected in protein-protein interaction network analysis

**Supplementary information**


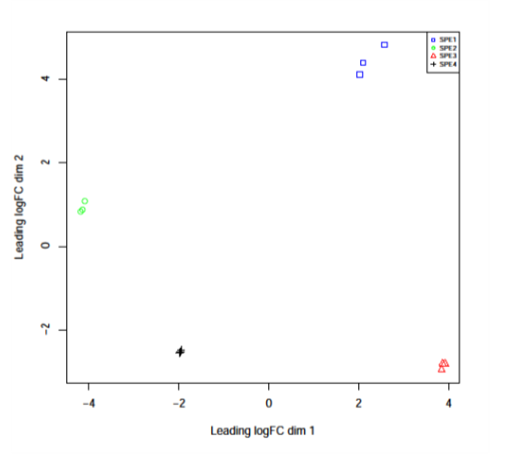


**Supplementary information, Fig. S1.**


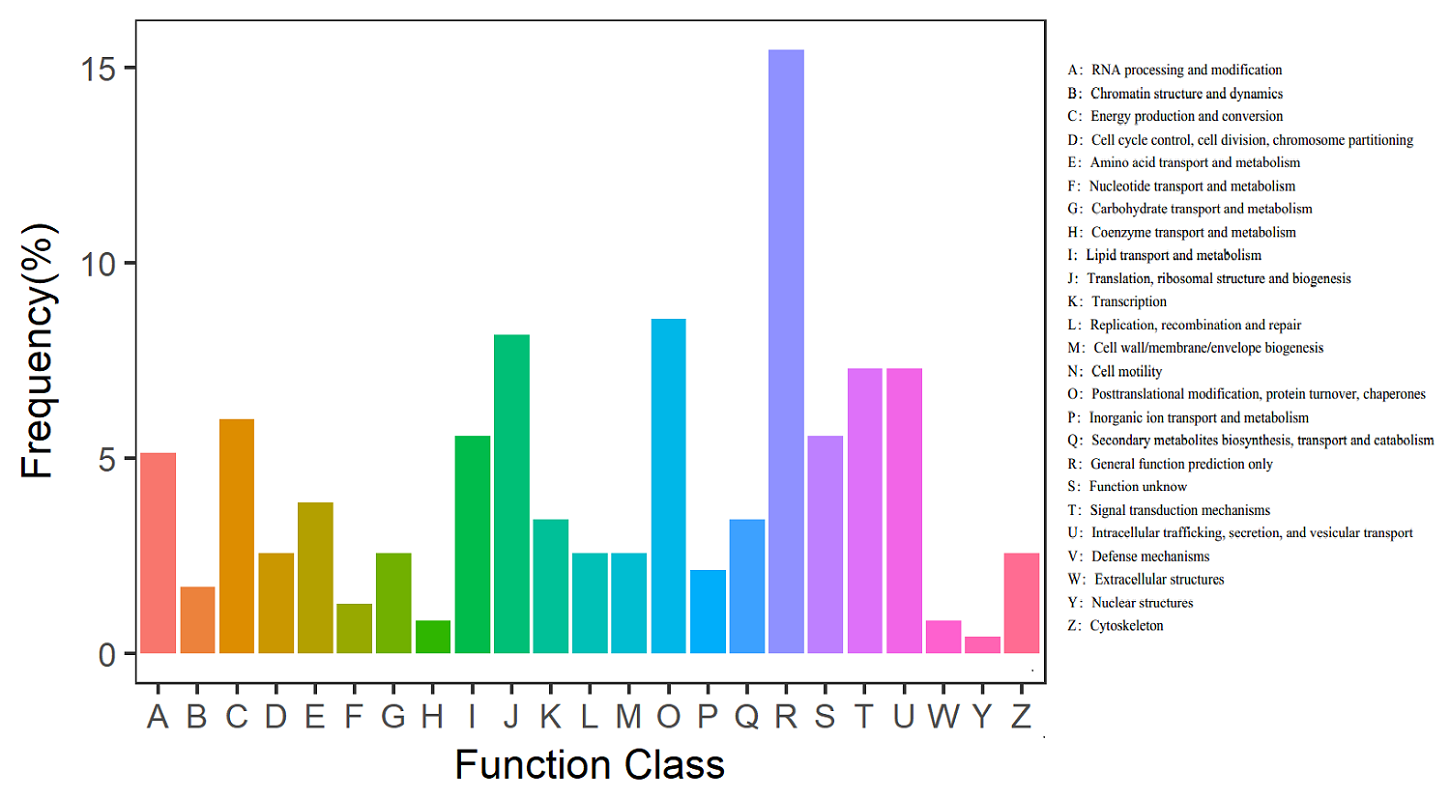


**Supplementary information, Fig. S2.**

**Supplementary information, Fig. S3.**


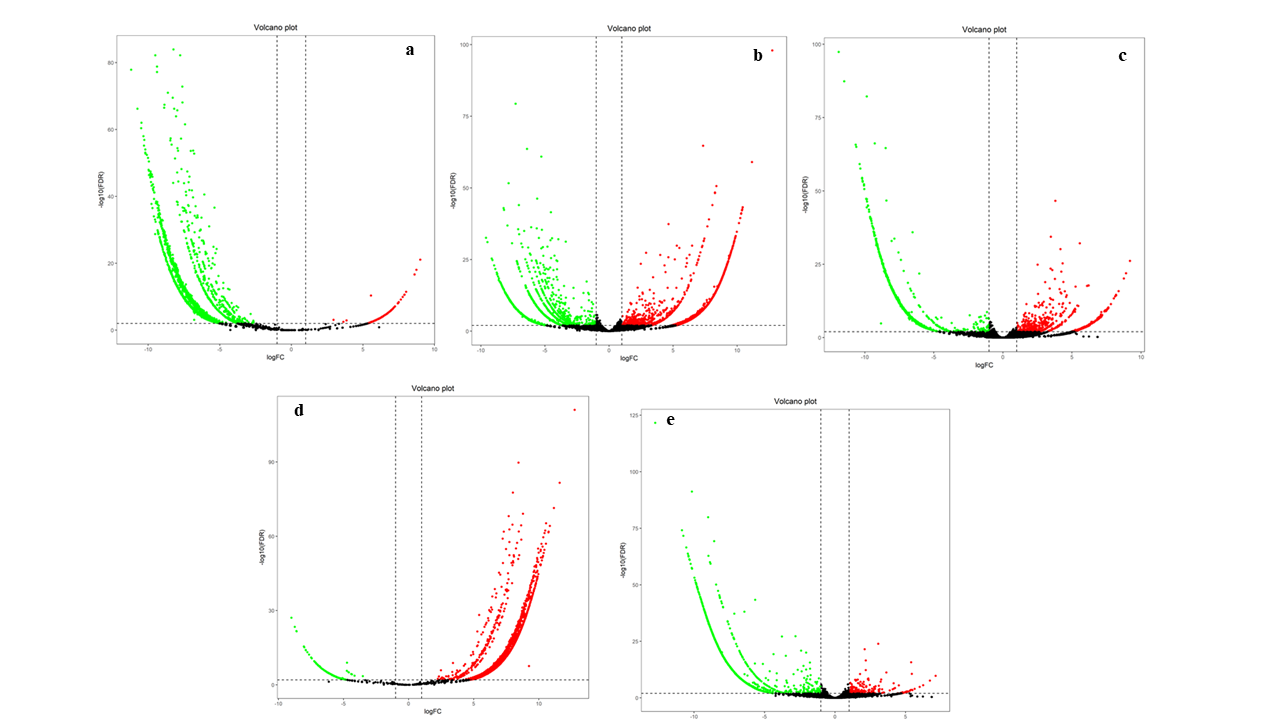


**Supplementary information, Fig. S4.**


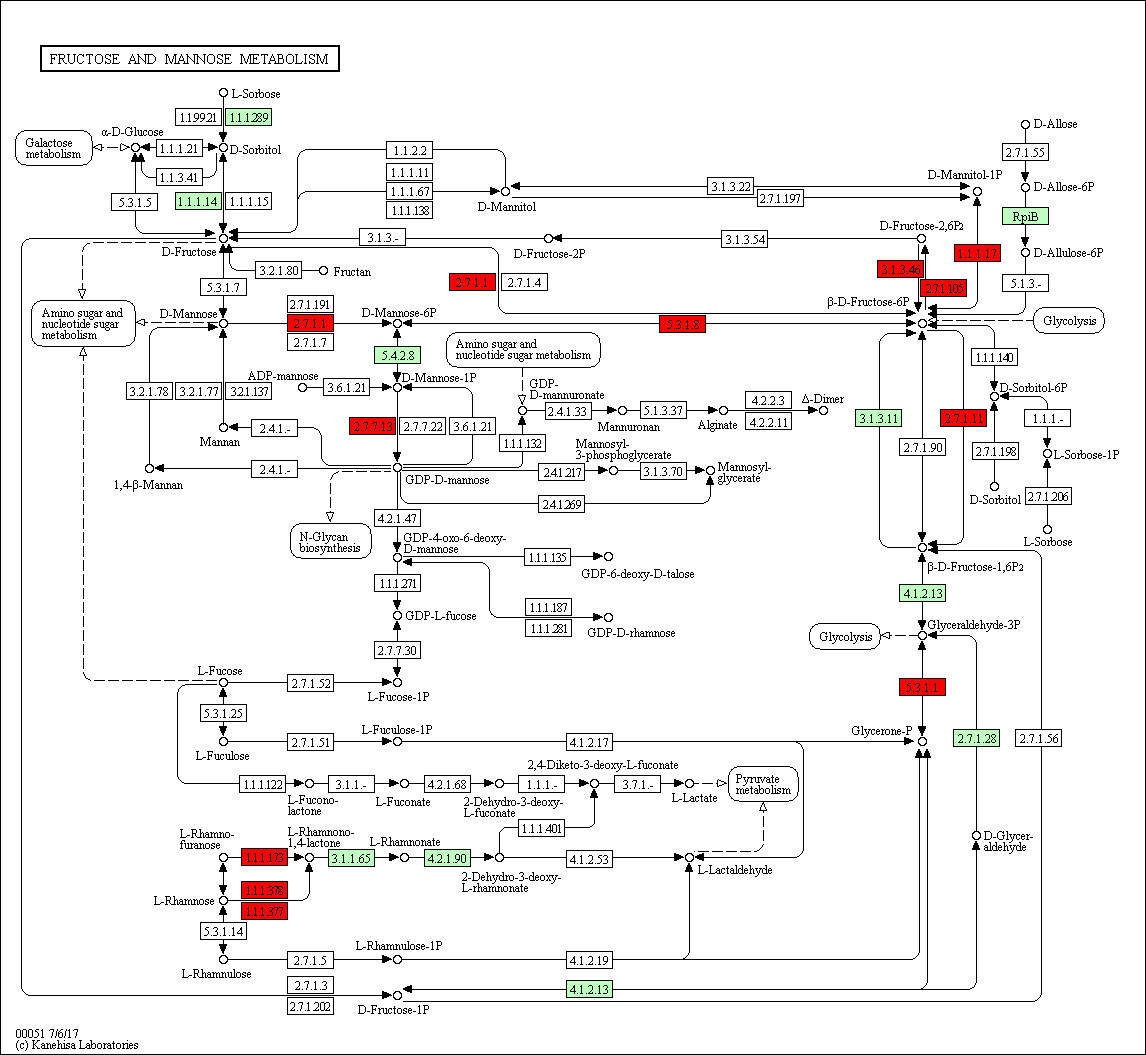


**Supplementary information, Fig. S5.**


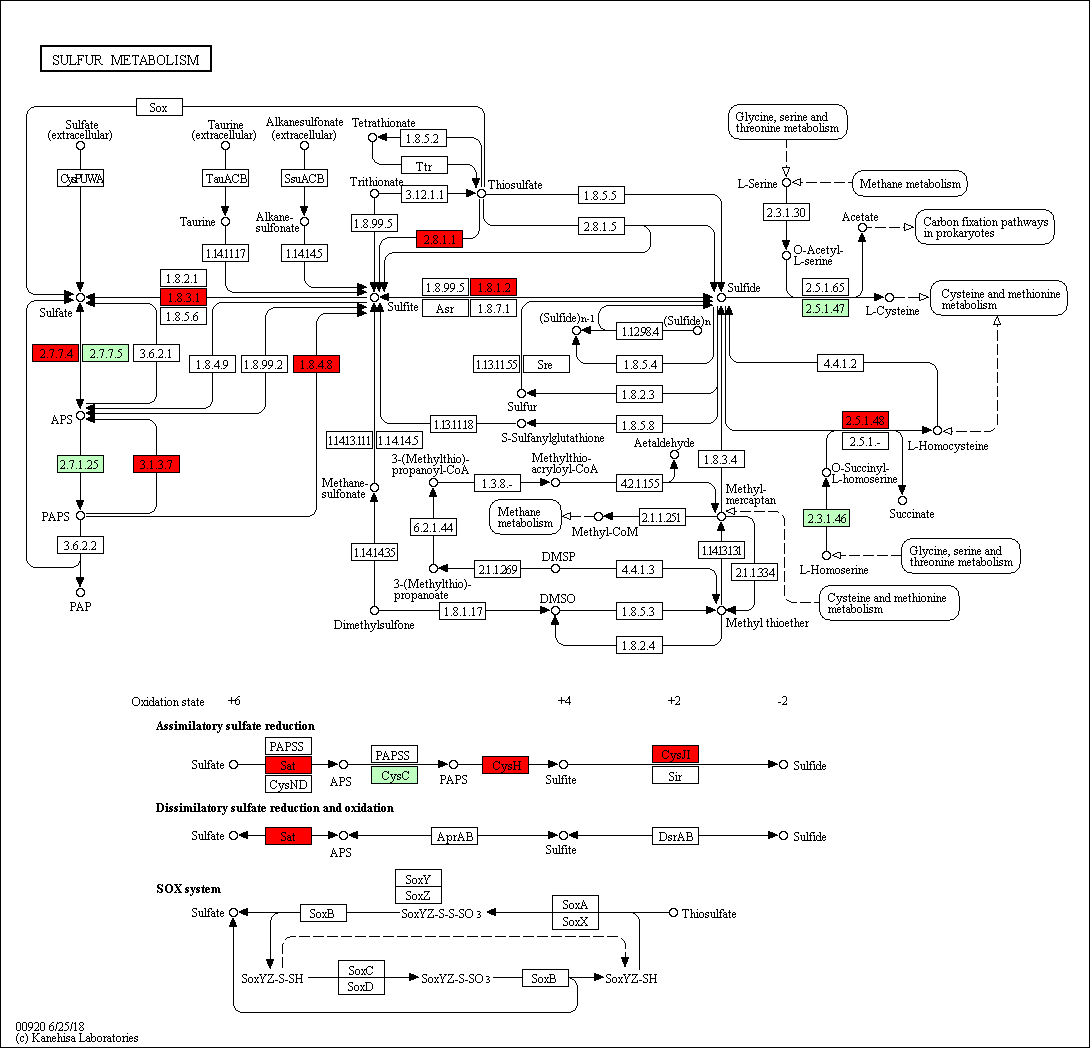


**Supplementary information, Fig. S6.**


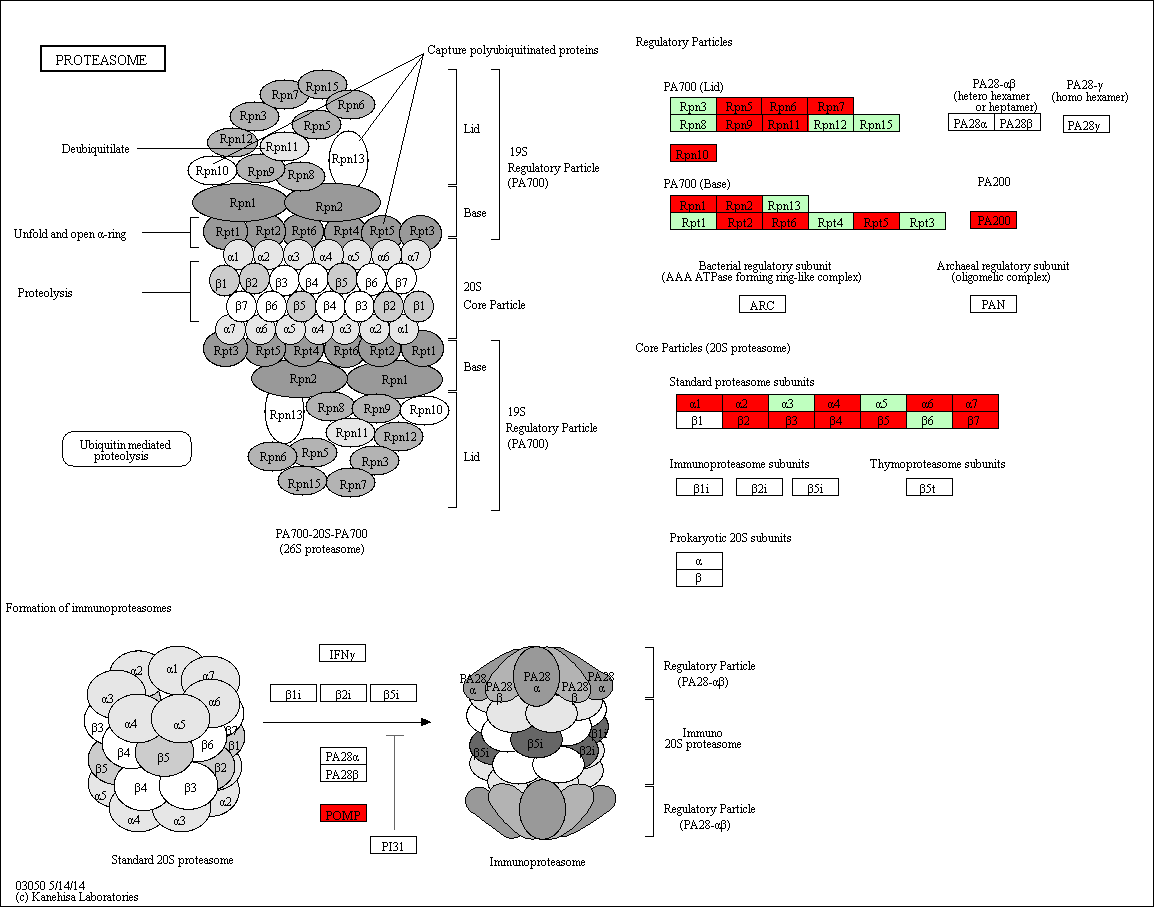


**Supplementary information, Fig. S7.**


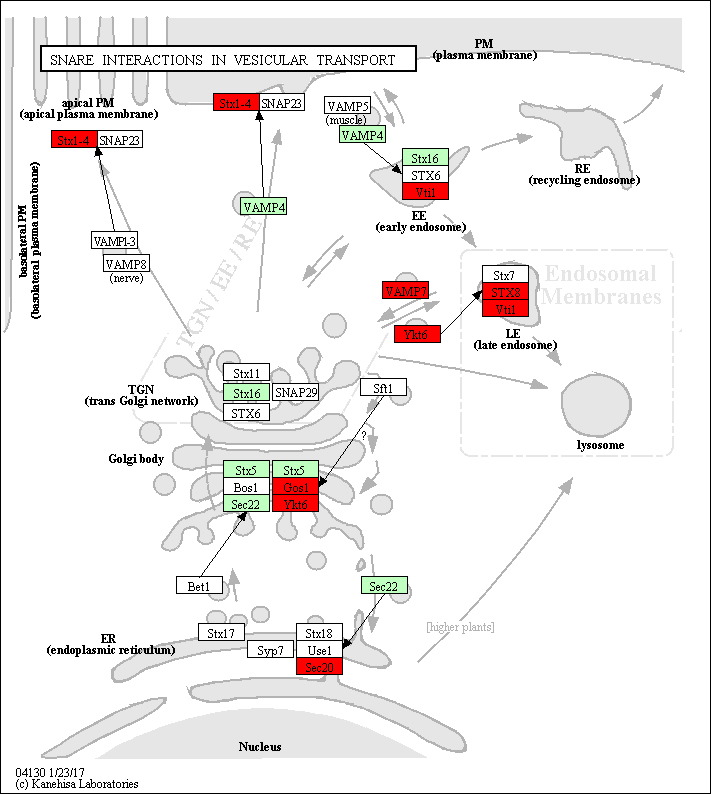


**Supplementary information, Fig. S8.**

**Supplementary information, Table S1.**

| **Gene** | **Description** | **Regulated** | | |
| --- | --- | --- | --- | --- |
|  | **Polyketides** | **SPE2** | **SPE3** | **SPE4** |
| Cluster-6084.0 | A0A179GXW7, Malonyl CoA-acyl carrier protein trans.. | Down |  |  |
| Cluster-7845.0 | A0A179HFX8, Alcohol dehydrogenase, zinc-containing | Down |  |  |
| Cluster-9540.0 | A0A179HGJ7, Zinc-binding dehydrogenase | Down |  |  |
| Cluster-5644.0 | A0A179H392, Polyketide synthase | Down |  |  |
| Cluster-5644.1 | A0A179H392, Polyketide synthase | Down |  |  |
| Cluster-5644.1 | A0A179H392, Polyketide synthase | Down |  |  |
| Cluster-6005.0 | A0A179HSY7, Nonribosomal siderophore peptide synthase | Down |  |  |
| Cluster-6406.0 | A0A179H502, Amidase | Down |  |  |
| Cluster-7555.0 | A0A179GJJ4, Zinc-binding dehydrogenase | Down |  |  |
| Cluster-9355.0 | A0A179H593, Cyclase/dehydrase family protein | Down |  |  |
| Cluster-7878.0 | A0A179GJS0, Protein TOXD | Down | Down |  |
| Cluster-5629.1 | Q2UH45, Uncharacterized protein | Down | Down | Down |
| Cluster-5972.0 | I8AD10, Alcohol dehydrogenase, class V | Down | Down | Down |
| Cluster-6333.0 | Q5VDF2, PksA | Down | Down | Down |
| Cluster-6655.0 | B8NL80, Fatty acid synthase beta subunit, putative | Down | Down | Down |
| Cluster-6835.0 | Q2UG06, Uncharacterized protein | Down | Down | Down |
| Cluster-7091.0 | I8U9N6, Uncharacterized protein | Down | Down | Down |
| Cluster-7644.0 | I8U3N1,3-oxoacyl-[acyl-carrier protein] reductase | Down | Down | Down |
| Cluster-7904.0 | B8MZZ4, Uncharacterized protein | Down | Down | Down |
| Cluster-8088.0 | I8TXN3, Quinone reductase | Down | Down | Down |
| Cluster-8189.0 | I8A836, Enoyl reductase domain of FAS1 | Down | Down | Down |
| Cluster-8231.0 | Q2UMH7, Uncharacterized protein | Down | Down | Down |
| Cluster-8231.0 | I8TH45, NADPH quinone reductase | Down | Down | Down |
| Cluster-8356.0 | B8NI04, AflC / pksA / pksL1 / polyketide synthase | Down | Down | Down |
| Cluster-8356.1 | B8NI04, AflC / pksA / pksL1 / polyketide synthase | Down | Down | Down |
| Cluster-8356.2 | Q5VDF2, PksA | Down | Down | Down |
| Cluster-8394.0 | I7ZMR3, Zinc-binding oxidoreductase | Down | Down | Down |
| Cluster-8878.0 | B8NI12, Hybrid PKS/NRPS enzyme, putative | Down | Down | Down |
| Cluster-9411.0 | I8A576, Polyketide synthase module | Down | Down | Down |
| Cluster-9411.1 | Q2UB00, Uncharacterized protein | Down | Down | Down |
| Cluster-9292.0 | A0A179HXF3, Alcohol dehydrogenase superfamily, zinc-containing | Down |  | Down |
| Cluster-9292.0 | A0A179HXF3, Alcohol dehydrogenase superfamily, zinc-containing | Down |  | Down |
| Cluster-7091.1 | I8U9N6, Uncharacterized protein |  | Down | Down |
| Cluster-8413.1 | B8NAC6, Quinone oxidoreductase, putative |  | Down | Down |
| **Genes** | **Amidases** |  |  |  |
| Cluster-5114.0 | A0A179HEL8, Amidase signature | Down |  |  |
| Cluster-6588.0 | A0A179H506, GPI transamidase component PIG-S | Down |  |  |
| Cluster-6406.0 | A0A179H502, Amidase | Down |  |  |
| **Gene** | **Description** | **Regulated** | | |
|  | **Amidases** | **SPE2** | **SPE3** | **SPE4** |
| Cluster-9107.0 | A0A179GZY2, Peptide-N4- | Down |  |  |
| Cluster-8716.0 | A0A179GTP5, Amidase signature domain-containingprotein | Down |  |  |
| Cluster-8387.0 | A0A179GMK1, Glutamyl-tRNA | Down |  |  |
| Cluster-8621.0 | A0A179GK75, Beta-alanine synthase | Down |  |  |
| Cluster-8354.0 | A0A179GFV2, N-acylethanolamine amidohydrolase | Down |  |  |
| Cluster-8094.0 | A0A179GFI4, Pyrazinamidase/nicotinamidase | Down |  |  |
| Cluster-8094.1 | A0A179GFI4, Pyrazinamidase/nicotinamidase | Down |  |  |
| Cluster-8716.1 | A0A179GDL3, Amidase signature domain-containingprotein | Down |  |  |
| Cluster-6230.0 | A0A179G2N5, Amidase | Down |  |  |
| Cluster-5114.0 | A0A179HEL8, Amidase signature | Down |  |  |
| Cluster-9686.0 | B8NVX6, Muramidase, putative | Down | Down | Down |
| Cluster-9189.0 | A0A0L1J869, Alkaline phytoceramidase | Down | Down | Down |
| Cluster-6476.0 | A0A0L1J4N3, Alkaline phytoceramidase | Down | Down | Down |
| Cluster-7361.0 | A0A179GKR1, Fatty-acid amide hydrolase | Down |  | Down |
|  | **Esterases** |  |  |  |
| Cluster-2557.1 | A0A179HC52,Purple acid phosphatase | Down |  |  |
| Cluster-3119.0 | A0A179HGH9,Phosphoinositide phospholipase C | Down |  |  |
| Cluster-4051.2 | A0A179GKD1,Phosphoinositide phospholipase C | Down |  |  |
| Cluster-5387.1 | A0A179HTD1,Acyl-CoA thioesterase | Down |  |  |
| Cluster-5589.0 | A0A1T3CI52,Thioesterase family protein | Down |  |  |
| Cluster-6190.0 | A0A179H0S0,Carboxylic ester hydrolase | Down |  |  |
| Cluster-6190.0 | A0A179H0S0,Carboxylic ester hydrolase | Down |  |  |
| Cluster-6774.0 | A0A0F7ZIQ1,Uncharacterized protein | Down |  |  |
| Cluster-7432.0 | A0A179GDA7,Ankyrin repeat protein | Down |  |  |
| Cluster-7634.0 | A0A179HGH8,Lariat debranching enzyme | Down |  |  |
| Cluster-8036.0 | A0A179HCI3,Phosphoethanolamine transferase class O | Down |  |  |
| Cluster-8136.0 | A0A179HHA0,Type I phosphodiesterase / nucleotide pyrophosphatase family protein | Down |  |  |
| Cluster-8507.0 | A0A179FQN0,Tat pathway signal sequence | Down |  |  |
| Cluster-8507.1 | A0A179FQN0,Tat pathway signal sequence | Down |  |  |
| Cluster-8507.1 | A0A179FQN0,Tat pathway signal sequence | Down |  |  |
| Cluster-8713.0 | A0A179GLQ8,Thioesterase superfamily protein | Down |  |  |
| Cluster-8920.0 | A0A0C2FPA0,tRNA ligase | Down |  |  |
| Cluster-9274.0 | A0A179H347,PLC-like phosphodiesterase | Down |  |  |
| Cluster-9315.0 | A0A179GVJ0,Carbohydrate esterase family 3 | Down |  |  |
| Cluster-5176.0 | Q2UGA2,Sphingomyelin phosphodiesterase | Down | Down | Down |
| Cluster-5615.1 | B8N3K0,Ser/Thr protein phosphatase family protein | Down | Down | Down |
| Cluster-5818.0 | Q2U2Y0,Endopolyphosphatase | Down | Down | Down |
| Cluster-6121.0 | A0A0D9NBJ7,Palmitoyl protein thioesterase | Down | Down | Down |
| **Gene** | **Description** | **Regulated** | | |
|  | **Esterases** | **SPE2** | **SPE3** | **SPE4** |
| Cluster-6333.0 | Q5VDF2,PksA | Down | Down | Down |
| Cluster-6767.0 | I8A3Y4, Cell division control protein/putative DNA repair exonuclease | Down | Down | Down |
| Cluster-6835.0 | Q2UG06, Uncharacterized protein | Down | Down | Down |
| Cluster-6912.0 | A0A0D9N5X2, Serine/threonine-protein phosphatase | Down | Down | Down |
| Cluster-7169.0 | A0A0D9MNT2, Calcineurin-like phosphoesterase | Down | Down | Down |
| Cluster-7720.0 | B8N652, PAF acetylhydrolase family protein | Down | Down | Down |
| Cluster-7759.0 | Q2UED0, Uncharacterized protein | Down | Down | Down |
| Cluster-8356.0 | B8NI04, AflC / pksA / pksL1 / polyketide synthase | Down | Down | Down |
| Cluster-8356.1 | B8NI04, AflC / pksA / pksL1 / polyketide synthase | Down | Down | Down |
| Cluster-8356.2 | Q5VDF2, PksA | Down | Down | Down |
| Cluster-8686.0 | B8MYF1, Lipase, putative | Down | Down | Down |
| Cluster-8776.0 | A0A0D9N3H8, PolyA polymerase central domain protein | Down | Down | Down |
| Cluster-5615.0 | Q2UK83, Uncharacterized protein | Down |  | Down |
| Cluster-5724.0 | A0A179H6J0, Glycerophosphoryl diester phosphodiesterase | Down |  | Down |
| Cluster-8801.0 | A0A0D9MYX8, Protein phosphatase methylesterase 1 | Down |  | Down |
| Cluster-9859.2 | A0A0D9MZX5, Cation efflux family protein |  | Down | Down |
| **Genes** | **Proteases** | **SPE2** | **SPE3** | **SPE4** |
| Cluster-10044.0 | A0A0P6J7Q6, Transitional endoplasmic reticulum ATPase | Down |  |  |
| Cluster-10545.0 | A0A2C5Z078, Uncharacterized protein | Down |  |  |
| Cluster-10545.1 | A0A179GTF0, Ubiquitinyl hydrolase 1 | Down |  |  |
| Cluster-5986.0 | A0A179H877, SnRNP assembly factor | Down |  |  |
| Cluster-5986.1 | A0A179GHN5, SnRNP assembly factor | Down |  |  |
| Cluster-6401.0 | A0A179GXF1, Ubiquitin carboxyl-terminal hydrolase | Down |  |  |
| Cluster-6616.0 | A0A179H511, Aspartyl protease domain-containing protein | Down |  |  |
| Cluster-7513.1 | A0A179GR97, Cysteine protease PalB | Down |  |  |
| Cluster-7513.1 | A0A179HEV0, Cysteine protease PalB | Down |  |  |
| Cluster-7823.0 | A0A179GBZ4, Eukaryotic aspartyl protease | Down |  |  |
| Cluster-8873.0 | A0A179HL28, COP9 signalosome complex subunit 5 | Down |  |  |
| Cluster-6984.1 | A0A0L0NDT3, LON peptidase N-terminal domain and RING finger protein 1 | Down | Down |  |
| Cluster-5723.0 | A0A0D9MTN8, Ulp1 protease family C-terminal catalytic domain protein | Down | Down | Down |
| Cluster-5763.0 | B8NS01, Aspartic endopeptidase | Down | Down | Down |
| Cluster-5980.0 | B8NVB0, Cysteine protease | Down | Down | Down |
| Cluster-6154.0 | B8NNS3, Ubiquitin carboxyl-terminal hydrolase | Down | Down | Down |
| Cluster-6779.0 | Q2UTS7, Uncharacterized protein | Down | Down | Down |
| Cluster-7022.0 | B8N3A1, ADAM family of metalloprotease ADM-B | Down | Down | Down |
| Cluster-7170.0 | B8NWT9, Aspartic-type endopeptidase, putative | Down | Down | Down |
| Cluster-7170.1 | B8NWT9, Aspartic-type endopeptidase, putative | Down | Down | Down |
| Cluster-7748.0 | I8U9S6, Uncharacterized protein | Down | Down | Down |
| Cluster-7826.0 | I8IU94, Cytosolic Ca2+-dependent cysteine protease, large subunit | Down | Down | Down |
| **Gene** | **Description** | **Regulated** | | |
|  | **Proteases** | **SPE2** | **SPE3** | **SPE4** |
| Cluster-7896.0 | B8NGC1, Lon protease homolog, mitochondrial | Down | Down | Down |
| Cluster-8007.0 | B8NLQ9, Lon protease homolog 2, peroxisomal | Down | Down | Down |
| Cluster-8373.0 | B8NKF3, Ubiquitin C-terminal hydrolase, putative | Down | Down | Down |
| Cluster-8627.0 | B8NFR0, Mitochondrial inner membrane AAA protease Yta12, putative | Down | Down | Down |
| Cluster-8910.0 | B8NS75, Yapsin, putative | Down | Down | Down |
| Cluster-9119.0 | I8TNH9, Ubiquitin carboxyl-terminal hydrolase | Down | Down | Down |
| Cluster-9221.2 | I8AB24, DNA damage inducible protein | Down | Down | Down |
| Cluster-9291.0 | I8U106, Ubiquitin-specific protease | Down | Down | Down |
| Cluster-9753.0 | Q2U6G4, Uncharacterized protein | Down | Down | Down |
| Cluster-9774.0 | A0A0D9MX53, Eukaryotic aspartyl protease | Down | Down | Down |
| **Gene** | **Lipases** |  |  |  |
| Cluster-4051.2 | A0A179GKD1, Phosphoinositide phospholipase C | Down |  |  |
| Cluster-3119.0 | A0A179HGH9, Phosphoinositide phospholipase C | Down |  |  |
| Cluster-6333.0 | Q5VDF2, PksA | Down | Down | Down |
| Cluster-8356.2 | Q5VDF2, PksA | Down | Down | Down |
| Cluster-6835.0 | Q2UG06, Uncharacterized protein | Down | Down | Down |
| Cluster-8356.0 | B8NI04, AflC / pksA / pksL1 / polyketide synthase | Down | Down | Down |
| Cluster-8356.1 | B8NI04, AflC / pksA / pksL1 / polyketide synthase | Down | Down | Down |
|  | **Chitinases** |  |  |  |
| Cluster-5693.0 | A0A0D9MQQ2, Glycosyl hydrolases family 18 | Down | Down | Down |
| Cluster-7899.1 | A0A024HW64, Chitinase 18-5 | Down |  |  |
| Cluster-7899.1 | A0A179HU36, Glycoside hydrolase family 18 | Down |  |  |
|  | **Cellulases** |  |  |  |
| Cluster-262.0 | A0A179I0H1, Cellulase family protein | Down |  |  |
| Cluster-8512.0 | A0A179HVM4, Exo-beta-1,3-glucanase | Down |  |  |
| **Genes** | **Glucanases** | **SPE2** | **SPE3** | **SPE4** |
| Cluster-4337.0 | A0A179HK19, Concanavalin A-like lectin/glucanase | Down |  |  |
| Cluster-4337.0 | A0A179HK19, Concanavalin A-like lectin/glucanase | Down |  |  |
| Cluster-8030.0 | A0A179HIZ8, Xylanase 3 | Down |  |  |
| Cluster-8381.0 | A0A179HVQ6, Endo-1,3-beta-glucanase Engl1 | Down |  |  |
| Cluster-8512.0 | A0A179HVM4, Exo-beta-1,3-glucanase | Down |  |  |
| Cluster-10594.0 | I8TSM5, SPRY domain-containing protein | Down | Down | Down |
| Cluster-6461.0 | B8N3J2, Endo-1,3-beta-glucanase Engl1 | Down | Down | Down |
| Cluster-6858.1 | Q2UEY5, Uncharacterized protein | Down | Down | Down |
| Cluster-7469.0 | I8AD97, Uncharacterized protein | Down | Down | Down |
| Cluster-8315.0 | I8A7F0, SPRY domain-containing protein | Down | Down | Down |
| Cluster-8315.0 | B8MZI6, Endosomal SPRY domain protein, putative | Down | Down | Down |
| Cluster-6858.0 | B8NH97, Lectin family integral membrane protein, putative | Down |  | down |
| **Genes** | **Description** | **Regulated** | | |
|  | **Glucan** | **SPE2** | **SPE3** | **SPE4** |
| Cluster-7809.0 | A0A179GSE2,1,3-beta-glucanosyltransferase | Down |  |  |
| Cluster-6133.0 | A0A179GRX2, Protein related to glucan 1, 4-alpha-glucosidase | Down |  |  |
| Cluster-4337.0 | A0A179HK19, Concanavalin A-like lectin/glucanase | Down |  |  |
| Cluster-4337.0 | A0A179HK19, Concanavalin A-like lectin/glucanase | Down |  |  |
| Cluster-8030.0 | A0A179HIZ8, Xylanase 3 | Down |  |  |
| Cluster-8381.0 | A0A179HVQ6, Endo-1,3-beta-glucanase Engl1 | Down |  |  |
| Cluster-8512.0 | A0A179HVM4, Exo-beta-1,3-glucanase | Down |  |  |
| Cluster-10594.0 | I8TSM5, SPRY domain-containing protein | Down | Down | Down |
| Cluster-5948.0 | B8MW03, Uncharacterized protein | Down | Down | Down |
| Cluster-6044.0 | I8TXP1, Glucan endo-1,3-beta-glucosidase eglC | Down | Down | Down |
| Cluster-6461.0 | B8N3J2, Endo-1,3-beta-glucanase Engl1 | Down | Down | Down |
| Cluster-6858.1 | Q2UEY5, Uncharacterized protein | Down | Down | Down |
| Cluster-7469.0 | I8AD97, Uncharacterized protein | Down | Down | Down |
| Cluster-8315.0 | I8A7F0, SPRY domain-containing protein | Down | Down | Down |
| Cluster-9650.0 | I8A653,1,3-beta-glucanosyltransferase | Down | Down | Down |
| Cluster-6858.0 | B8NH97, Lectin family integral membrane protein, putative | Down |  | Down |
| **Genes** | **GPI** |  |  |  |
| Cluster-6826.0 | A0A179GF44, GPI mannosyltransferase | Down |  |  |
| Cluster-6826.0 | A0A179GF44, GPI mannosyltransferase | Down |  |  |
| Cluster-3738.2 | A0A179HU98, N-acetylglucosaminyl transferase component Gpi1 | Down |  |  |
| Cluster-6588.0 | A0A179H506, GPI transamidase component PIG-S | Down |  |  |
| Cluster-7407.0 | A0A179HU40, GPI anchored protein | Down |  |  |
| Cluster-8032.0 | A0A179HSM3, GPI anchored protein | Down |  |  |
| Cluster-8036.0 | A0A179HCI3, Phosphoethanolamine transferase class O | Down |  |  |
| Cluster-8422.0 | A0A151GE42, GPI-GlcNAc transferase complex | Down |  |  |
| Cluster-6968.0 | A0A179GNR8, Gpi-anchored cell surface glycoprotein | Down |  |  |
| Cluster-5189.0 | A0A179GPI3, L-fucose permease | Down |  |  |
| Cluster-9968.0 | A0A179GE82, Subunit P of phosphatidylinositol N-acetylglucosaminyltransferase | Down | Down |  |
| Cluster-10695.0 | Q2UGA5, Uncharacterized protein | Down | Down | Down |
| Cluster-6069.0 | B8NEX1, GPI-anchor biosynthesis protein | Down | Down | Down |
| Cluster-7438.1 | B8NR36, GPI anchored glycoprotein, putative | Down | Down | Down |
| Cluster-7898.0 | I8A801, GPI mannosyltransferase 2 | Down | Down | Down |
| Cluster-8196.0 | B8NMZ7, GPI anchored cell wall protein, putative | Down | Down | Down |
| Cluster-8364.0 | B8MZR2, Glycan biosynthesis protein | Down | Down | Down |
| Cluster-9380.1 | B8MYF3, Uncharacterized protein | Down | Down | Down |
| Cluster-6112.1 | B8NSI9, Mannosyltransferase | Down |  | Down |
| **Genes** | **Description** | **Regulated** | | |
|  | **P450** | **SPE2** | **SPE3** | **SPE4** |
| Cluster-6775.1 | A0A179HCU6, Cytochrome P450 | Down |  |  |
| Cluster-9599.0 | A0A179GM99, Uncharacterized protein | Down |  |  |
| Cluster-4738.0 | A0A179HPG3, Cytochrome P450 52A11 | Down |  |  |
| Cluster-4738.0 | A0A179GW37, Cytochrome P450 52A11 | Down |  |  |
| Cluster-6645.0 | A0A179GGA6, Cytochrome P450 | Down |  |  |
| Cluster-6645.0 | W9CLP6, Putative cytochrome p450 | Down |  |  |
| Cluster-10339.0 | A0A179G171, Linoleate diol synthase | Down |  |  |
| Cluster-10339.0 | A0A179HSZ2, Linoleate diol synthase | Down |  |  |
| Cluster-10340.0 | A0A179GG21, Cytochrome P450 alkane hydroxylase | Down |  |  |
| Cluster-5908.0 | A0A179HS32, Benzoate 4-monooxygenase | Down |  |  |
| Cluster-6808.0 | A0A179HVY0, Sulfite reductase flavoprotein alpha-component | Down |  |  |
| Cluster-7112.1 | A0A179GXD3, Cytochrome P450 | Down |  |  |
| Cluster-8197.0 | A0A179H948, Cytochrome P450 family protein | Down |  |  |
| Cluster-9244.1 | A0A179H4A0, Cytochrome P450 52E1 | Down |  |  |
| Cluster-9244.1 | A0A179H4A0, Cytochrome P450 52E1 | Down |  |  |
| Cluster-8280.0 | A0A179FKC6, Cytochrome P450 | Down | Down |  |
| Cluster-5498.0 | I7ZSL3, NADPH--cytochrome P450 reductase | Down | Down | Down |
| Cluster-6362.0 | B8NYC3, Glutathione S-transferase family protein, putative | Down | Down | Down |
| Cluster-6621.0 | A0A0D9N965, Cytochrome P450 | Down | Down | Down |
| Cluster-6797.0 | B8N8Y9, NADPH--cytochrome P450 reductase | Down | Down | Down |
| Cluster-6819.0 | B8NKB3, Cytochrome P450 alkane hydroxylase, putative | Down | Down | Down |
| Cluster-7028.0 | B8N9G1, Aldehyde dehydrogenase, putative | Down | Down | Down |
| Cluster-7474.0 | B8NHY2, AflV/ cypX/ cytochrome P450 monooxygenase | Down | Down | Down |
| Cluster-7575.0 | B8N0G5, Cytochrome p450, putative | Down | Down | Down |
| Cluster-7895.0 | I8A827, Cytochrome protein | Down | Down | Down |
| Cluster-8585.0 | B8N5A3, Cytochrome P450, putative | Down | Down | Down |
| Cluster-8599.0 | I8THZ9, Cytochrome protein | Down | Down | Down |
| Cluster-8690.0 | B8NYC5, Cytochrome P450 oxidoreductase, putative | Down | Down | Down |
| Cluster-8768.0 | A0A1S9E1P5, Cytochrome P450 | Down | Down | Down |
| Cluster-9134.0 | B8NBF2, Cytochrome P450 family protein, putative | Down | Down | Down |
| Cluster-9345.0 | I8U156, Cytochrome protein | Down | Down | Down |
| Cluster-9530.0 | B8NXA4, Benzoate 4-monooxygenase cytochrome P450, putative | Down | Down | Down |
| Cluster-9627.0 | A0A0D9MWU8, FAD binding domain protein | Down | Down | Down |
| Cluster-9909.0 | Q2UIY1, Uncharacterized protein | Down | Down | Down |
| Cluster-9909.1 | B8N0G4, Cytochrome P450, putative | Down | Down | Down |
| Cluster-5498.2 | R8BI90, NADPH--cytochrome P450 reductase | Down |  | Down |
| Cluster-6586.0 | Q2TXT8, Uncharacterized protein | Down |  | Down |
| Cluster-9134.1 | Q2TZU9, Uncharacterized protein | Down |  | Down |
| **Genes** | **Membrane proteins** | **Regulated** | | |
| Cluster-5478.0 | A0A179GY80, Iron-regulated transporter | Down |  |  |
| Cluster-6209.1 | A0A179GW32, Pheromone-regulated membrane protein | Down |  |  |
| Cluster-8659.0 | A0A2C5YYV4, Uncharacterized protein | Down |  |  |
| Cluster-192.0 | A0A179HVN4, Ascus development protein 3 | Down |  |  |
| Cluster-5107.0 | A0A179HQX4, GTPase-activator protein for ras-like GTPase containing protein | Down |  |  |
| Cluster-5289.1 | A0A179GID1, Amino-acid permease inda1 | Down |  |  |
| Cluster-5431.0 | A0A179GMA0, MFS sugar transporter | Down |  |  |
| Cluster-6434.0 | A0A179GDP9, Amino acid transporter | Down |  |  |
| Cluster-6446.0 | A0A179GH45, Trk family potassium uptake protein | Down |  |  |
| Cluster-6446.0 | A0A0F8A280, Uncharacterized protein | Down |  |  |
| Cluster-6756.0 | A0A179GUD8, NCS1 nucleoside transporter family protein | Down |  |  |
| Cluster-6846.0 | A0A0L0N8S8, Putative chromate transport protein | Down |  |  |
| Cluster-7265.0 | A0A179GX70, Phosphate transporter | Down |  |  |
| Cluster-7265.1 | A0A179GX70, Phosphate transporter | Down |  |  |
| Cluster-7414.1 | A0A179HJ59, General amino-acid permease GAP1 | Down |  |  |
| Cluster-7414.2 | A0A179HJ59, General amino-acid permease GAP1 | Down |  |  |
| Cluster-7432.0 | A0A179GDA7, Ankyrin repeat protein | Down |  |  |
| Cluster-7616.1 | A0A179HMA5, Phosphate transporter | Down |  |  |
| Cluster-7662.0 | A0A179GU17, Sugar porter | Down |  |  |
| Cluster-7662.0 | A0A179GU17, Sugar porter | Down |  |  |
| Cluster-7732.0 | A0A179I086, ZIP metal ion transporter | Down |  |  |
| Cluster-8384.0 | A0A179HEZ8, Sugar porter | Down |  |  |
| Cluster-8555.0 | A0A179HQX4, GTPase-activator protein for ras-like GTPase containing protein | Down |  |  |
| Cluster-8565.0 | A0A179HXR0, Uncharacterized protein | Down |  |  |
| Cluster-8765.0 | A0A179GT23, Sulfate permease | Down |  |  |
| Cluster-8836.3 | A0A179GEQ2, Ctr copper transporter family protein | Down |  |  |
| Cluster-8923.0 | A0A179FWA5, MFS phospholipid transporter | Down |  |  |
| Cluster-8950.0 | A0A179GHQ8, Uracil permease | Down |  |  |
| Cluster-8950.0 | A0A179GHQ8, Uracil permease | Down |  |  |
| Cluster-9390.0 | A0A179HDU1, Quinate permease | Down |  |  |
| Cluster-9390.0 | A0A179HDU1, Quinate permease | Down |  |  |
| Cluster-9489.0 | A0A179FRW1, Urea transport protein | Down |  |  |
| Cluster-192.0 | A0A179GLU6, Ascus development protein | Down |  |  |
| Cluster-5195.0 | A0A179HCN1, Hexose carrier protein | Down | Down |  |
| Cluster-10089.0 | A0A0D9N5K7, Amino acid permease | Down | Down | Down |
| Cluster-10145.0 | I8IC73, Amino acid transporter | Down | Down | Down |
| Cluster-10233.0 | B8NKK0, Ctr copper transporter, putative | Down | Down | Down |
| Cluster-10297.0 | A0A1F7ZV76, V-type proton ATPase proteolipid subunit | Down | Down | Down |
| Cluster-10407.0 | A0A0D9N8T6, Amino acid permease | Down | Down | Down |
| Cluster-5155.0 | A0A0M9D7H4, Branched-chain amino acid transport system carrier protein | Down | Down | Down |
| **Genes** | **Membrane proteins** | **Regulated** | | |
| Cluster-5486.0 | A0A087EP12, Uncharacterized protein | Down | Down | Down |
| Cluster-5652.0 | A0A0D9N834, Amino acid permease | Down | Down | Down |
| Cluster-5911.0 | B8NW99, Amino acid transporter, putative | Down | Down | Down |
| Cluster-5961.0 | Q2UHC3, Uncharacterized protein | Down | Down | Down |
| Cluster-6041.0 | B8NRN9, Choline transport protein, putative | Down | Down | Down |
| Cluster-6280.0 | I7ZTZ8, H+/oligopeptide symporter | Down | Down | Down |
| Cluster-6280.1 | Q2TX98, Uncharacterized protein | Down | Down | Down |
| Cluster-6872.0 | I8A3S8, Putative transporter | Down | Down | Down |
| Cluster-7208.0 | I7ZQV8, Amino acid transporter | Down | Down | Down |
| Cluster-7329.0 | B8MX22, Mitochondrial carrier protein, putative | Down | Down | Down |
| Cluster-7434.0 | I8IRR4, Nucleoside transporter | Down | Down | Down |
| Cluster-7632.0 | B8N3B7, V-type proton ATPase subunit a | Down | Down | Down |
| Cluster-7632.0 | B8N3B7, V-type proton ATPase subunit a | Down | Down | Down |
| Cluster-8080.0 | A0A0F0IN39, Sugar and other transporter | Down | Down | Down |
| Cluster-8098.0 | A0A1F8AF16, Uncharacterized protein | Down | Down | Down |
| Cluster-8156.0 | A0A0L1J7X1, Molybdenum cofactor biosynthesis protein Gephyrin | Down | Down | Down |
| Cluster-8169.0 | B8N052, Ctr copper transporter family protein | Down | Down | Down |
| Cluster-8211.0 | A0A0D9MUM0, POT family protein | Down | Down | Down |
| Cluster-8211.1 | I7ZNW4, H+/oligopeptide symporter | Down | Down | Down |
| Cluster-8211.1 | B8NXG7, Oligopeptide transporter, putative | Down | Down | Down |
| Cluster-8331.0 | I8A849, Amino acid transporter | Down | Down | Down |
| Cluster-8358.0 | B8NDG0, Bicyclomycin resistance protein, putative | Down | Down | Down |
| Cluster-8358.1 | B8NDG0, Bicyclomycin resistance protein, putative | Down | Down | Down |
| Cluster-8525.0 | B8NR56, V-type proton ATPase proteolipid subunit | Down | Down | Down |
| Cluster-8758.0 | Q2UHF7, Uncharacterized protein | Down | Down | Down |
| Cluster-8758.1 | I8TL96, Amino acid transporter | Down | Down | Down |
| Cluster-8796.0 | A0A0D9MRF9, Sugar and other transporter | Down | Down | Down |
| Cluster-9026.0 | B8MZ34, Mitochondrial F1F0-ATP synthase g subunit, putative | Down | Down | Down |
| Cluster-9090.0 | A0A0D9N3X7, Sugar and other transporter | Down | Down | Down |
| Cluster-9143.0 | I8IK89, V-type proton ATPase subunit | Down | Down | Down |
| Cluster-9168.0 | B8NL23, Sugar transporter, putative | Down | Down | Down |
| Cluster-9859.0 | A0A0F0I1K7, Cation efflux family protein | Down | Down | Down |
| Cluster-9859.1 | I8TYQ6, Zn2+ transporter ZNT1 | Down | Down | Down |
| Cluster-9963.0 | Q2UJ08, Uncharacterized protein | Down | Down | Down |
| Cluster-9963.1 | B8N0J3, CorA family metal ion transporter, putative | Down | Down | Down |
| Cluster-5166.0 | A0A0F0I4Q8, Stretch-activated Ca2+-permeable channel component | Down |  | Down |
| Cluster-5706.0 | Q2U189, Uncharacterized protein | Down |  | Down |
| Cluster-8778.0 | I8A2U3, Zinc transporter | Down |  | Down |
| Cluster-9859.2 | A0A0D9MZX5, Cation efflux family protein |  | Down | Down |
| Cluster-6564.0 | Q2U422, Uncharacterized protein |  |  | Down |
| Cluster-9659.0 | A0A0F0I635, Mitochondrial carrier protein |  |  | Down |
| **Genes** | **Sporulation** | **Regulated** | | |
| Cluster-2133.1 | A0A179HI08, Sporulation-specific protein | Down |  |  |
| Cluster-4509.1 | A0A179HX56, Spo7-like protein | Down |  |  |
| Cluster-6039.0 | A0A179HCL8,5-azacytidine resistance protein azr1 | Down | Up |  |
| Cluster-7510.0 | Q2UC20, Uncharacterized protein | Down | Down | Down |
|  | **Melanogenesis** |  |  |  |
| Cluster-7739.0 | A0A179H3B5, cAMP-dependent protein kinase type 2 | Down | Up | Up |
| Cluster-6021.1 | Q2UCG4, Uncharacterized protein | Down | Down | Down |
| Cluster-6021.2 | Q2UCG4, Uncharacterized protein | Down | Down | Down |
| Cluster-5303.0 | A0A0D9MRC5, MAGE family protein | Down | Down | Down |
| Cluster-10013.0 | B8N223, Mitogen-activated protein kinase |  | Down | Down |
|  | **Toxin** |  |  |  |
| Cluster-9806.0 | A0A179HAP5, Zeta toxin family protein | Down |  |  |
| Cluster-6333.0 | Q5VDF2, PksA | Down | Down | Down |
| Cluster-8356.2 | Q5VDF2, PksA | Down | Down | Down |
| Cluster-6835.0 | Q2UG06, Uncharacterized protein | Down | Down | Down |
| Cluster-9644.0 | Q2NNG0, AFLR | Down | Down | Down |
| Cluster-7579.0 | I7ZW85, Uncharacterized protein | Down | Down | Down |
| Cluster-6655.0 | B8NL80, Fatty acid synthase beta subunit, putative | Down | Down | Down |
| Cluster-8356.0 | B8NI04, AflC / pksA / pksL1 / polyketide synthase | Down | Down | Down |
| Cluster-8356.1 | B8NI04, AflC / pksA / pksL1 / polyketide synthase | Down | Down | Down |
| Cluster-10013.0 | B8N223, Mitogen-activated protein kinase |  | Down | Down |
|  | **Mycotoxin** |  |  |  |
| Cluster-7579.0 | I7ZW85, Uncharacterized protein | Down | Down | Down |
|  | **Detoxification** |  |  |  |
| Cluster-10339.0 | A0A179G171, Linoleate diol synthase | Down |  |  |
| Cluster-10339.0 | A0A179HSZ2, Linoleate diol synthase | Down |  |  |
| Cluster-10339.0 | A0A2C6A6C3, Uncharacterized protein | Down |  |  |
| Cluster-10607.0 | Q2UGZ1, Peroxidase | Down | Down | Down |
| Cluster-5901.0 | B8NN64,4-carboxymuconolactone decarboxylase, putative | Down | Down | Down |
| Cluster-6493.0 | B8N9G4, Mitochondrial peroxiredoxin Prx1, putative | Down | Down | Down |
| Cluster-6602.0 | B8MZ48, Glutathione oxidoreductase Glr1, putative | Down | Down | Down |
| Cluster-7392.0 | I7ZXW6, Uncharacterized protein | Down | Down | Down |
| Cluster-8216.0 | B8NTF4, /TSA family thioredoxin peroxidase, putative | Down | Down | Down |
| Cluster-6601.0 | B8NQM9, Uncharacterized protein | Down |  | Down |
|  | **Virulence** |  |  |  |
| Cluster-3852.0 | A0A179GQ24, CAS1 protein | Down | Up | Up |
| Cluster-10704.1 | A0A1S9DI86, Uncharacterized protein | Down | Down | Down |
| Cluster-6303.0 | A0A179GCZ8, Cell surface protein | Down |  |  |
| Cluster-6478.1 | A0A179GIV1, LysM domain-containingprotein | Down |  |  |
| Cluster-9228.0 | A0A0D9MMV0, Lysophospholipase NTE1 | Down | Down | Down |
| Cluster-7702.0 | I8A662, Pectate lyase | Down | Down | Down |
| **Genes** | **Effector** | **Regulated** | | |
| Cluster-6543.2 | A0A179GX33, Dynamin family protein | Down | Up | Up |
| Cluster-7741.0 | A0A179GJ91, Vacuolar sorting protein 1 | Down |  |  |
| Cluster-8730.1 | A0A0F0IIW7, Dynamin central region | Down | Down | Down |
| Cluster-7437.0 | A0A0D9MUP9, Dynamin central region | Down | Down | Down |
| Cluster-8730.0 | B8NX83, Dynamin-like GTPase Dnm1, putative | Down | Down | Down |
|  | **Pathogen** |  |  |  |
| Cluster-7574.0 | A0A179GQI1, Actin-related protein 2/3 complex subunit 5 | Down |  |  |
| Cluster-7930.0 | A0A179GGY1, Sgs | Down |  |  |
| Cluster-9858.0 | B8N3W5, Actin-related protein 2/3 complex subunit 5 | Down | Down | Down |
| Cluster-5719.0 | A0A0P7J0X4, Chaperone protein ClpB | Down | Down | Down |
| Cluster-6125.0 | Q2UJ67, Actin-related protein 2/3 complex subunit 3 | Down | Down | Down |
|  | **Secreted proteins** |  |  |  |
| Cluster-9574.0 | A0A179GQM7, Proteinrelated to secreted protein-sviceus | Down | Down | Up |
| Cluster-7445.0 | A0A179HD86, Secreted protein | Down | Up | Up |
| Cluster-8800.0 | A0A179H3C1, Secreted protein NIS1 | Down |  |  |
| Cluster-8953.0 | A0A179HJ41, Conserved secreted protein | Down |  |  |
| Cluster-6219.0 | I8AD83, Cysteine-rich secreted protein | Down | Down | Down |
| Cluster-9656.0 | A0A224Y3C5, Putative secreted protein | Down | Down | Down |

**Supplementary information, Table S2.**

| **GO.ID** | **Term** | **DEGs size** | **Size** | **RF** | **P value** | **FDR** |
| --- | --- | --- | --- | --- | --- | --- |
|  | **Biological process** |  |  |  |  |  |
| GO:0044260 | Cellular macromolecule metabolic process | 1516 | 4259 | 0.782 | 0.036 | 0.046 |
| GO:0071840 | Cellular component organization or bioge... | 953 | 2625 | 0.798 | 0.017 | 0.033 |
| GO:0006996 | Organelle organization | 552 | 1493 | 0.812 | 0.020 | 0.036 |
| GO:0044085 | Cellular component biogenesis | 491 | 1321 | 0.817 | 0.019 | 0.036 |
| GO:0051641 | Cellular localization | 405 | 1093 | 0.814 | 0.038 | 0.047 |
| GO:1902589 | Single-organism organelle organization | 362 | 965 | 0.824 | 0.025 | 0.037 |
| GO:0051649 | Establishment of localization in cell | 340 | 902 | 0.828 | 0.023 | 0.036 |
| GO:0007049 | Cell cycle | 301 | 789 | 0.838 | 0.016 | 0.033 |
| GO:0046907 | Intracellular transport | 291 | 754 | 0.848 | 0.010 | 0.031 |
| GO:0034660 | ncrna metabolic process | 288 | 706 | 0.896 | 0.000 | 0.013 |
|  | **Cellular component** |  |  |  |  |  |
| GO:0005623 | Cell | 3106 | 8775 | 0.773 | 0.000 | 0.007 |
| GO:0044464 | Cell part | 3098 | 8758 | 0.772 | 0.000 | 0.007 |
| GO:0005737 | Cytoplasm | 2228 | 6282 | 0.774 | 0.042 | 0.045 |
| GO:0044444 | Cytoplasmic part | 1614 | 4415 | 0.798 | 0.001 | 0.007 |
| GO:0005634 | Nucleus | 1150 | 3110 | 0.807 | 0.001 | 0.008 |
| GO:0032991 | Macromolecular complex | 957 | 2535 | 0.824 | 0.000 | 0.007 |
| GO:0043234 | Protein complex | 724 | 1910 | 0.828 | 0.001 | 0.007 |
| GO:0031090 | Organelle membrane | 612 | 1656 | 0.807 | 0.026 | 0.039 |
| GO:0031974 | Membrane-enclosed lumen | 567 | 1510 | 0.820 | 0.009 | 0.021 |
| GO:0044428 | Nuclear part | 566 | 1502 | 0.823 | 0.007 | 0.020 |
|  | **Molecular function** |  |  |  |  |  |
| GO:0015631 | Tubulin binding | 50 | 107 | 1.050 | 0.004 | 0.048 |
| GO:0016765 | Transferase activity, transferring alkyl... | 48 | 108 | 0.999 | 0.014 | 0.048 |
| GO:0008017 | Microtubule binding | 40 | 85 | 1.058 | 0.008 | 0.048 |
| GO:0050661 | NADP binding | 40 | 93 | 0.967 | 0.040 | 0.048 |
| GO:0061630 | Ubiquitin protein ligase activity | 33 | 75 | 0.989 | 0.042 | 0.048 |
| GO:0043022 | Ribosome binding | 16 | 30 | 1.199 | 0.022 | 0.048 |
| GO:0051087 | Chaperone binding | 15 | 29 | 1.163 | 0.035 | 0.048 |
| GO:0016408 | C-acyltransferase activity | 14 | 22 | 1.430 | 0.004 | 0.048 |
| GO:0019783 | Ubiquitin-like protein-specific protease... | 14 | 24 | 1.311 | 0.012 | 0.048 |
| GO:0004298 | Threonine-type endopeptidase activity | 13 | 19 | 1.538 | 0.002 | 0.046 |

**Supplementary information, Table S3.**

| **GO.ID** | **Term** | **DEGs size** | **Size** | **RF** | **P value** | **FDR** |
| --- | --- | --- | --- | --- | --- | --- |
|  | **Biological process** |  |  |  |  |  |
| GO:0044711 | Single-organism biosynthetic process | 463 | 1915 | 0.822 | 0.023 | 0.038 |
| GO:0051234 | Establishment of localization | 462 | 1911 | 0.822 | 0.023 | 0.038 |
| GO:0006810 | Transport | 456 | 1871 | 0.829 | 0.014 | 0.028 |
| GO:1902578 | Single-organism localization | 392 | 1614 | 0.826 | 0.028 | 0.041 |
| GO:0044765 | Single-organism transport | 368 | 1515 | 0.826 | 0.033 | 0.044 |
| GO:0044085 | Cellular component biogenesis | 323 | 1321 | 0.831 | 0.033 | 0.044 |
| GO:0051641 | Cellular localization | 275 | 1093 | 0.856 | 0.013 | 0.027 |
| GO:0051649 | Establishment of localization in cell | 237 | 902 | 0.893 | 0.002 | 0.017 |
| GO:0006396 | RNA processing | 221 | 842 | 0.892 | 0.004 | 0.019 |
| GO:0046907 | Intracellular transport | 205 | 754 | 0.924 | 0.001 | 0.010 |
|  | **Cellular component** |  |  |  |  |  |
| GO:0044464 | Cell part | 2004 | 8758 | 0.773 | 0.000 | 0.002 |
| GO:0005737 | Cytoplasm | 1479 | 6282 | 0.796 | 0.000 | 0.002 |
| GO:0043227 | Membrane-bounded organelle | 1333 | 5627 | 0.801 | 0.000 | 0.002 |
| GO:0044444 | Cytoplasmic part | 1059 | 4415 | 0.811 | 0.000 | 0.003 |
| GO:0032991 | Macromolecular complex | 624 | 2535 | 0.832 | 0.001 | 0.005 |
| GO:0043234 | Protein complex | 462 | 1910 | 0.817 | 0.021 | 0.031 |
| GO:0031090 | Organelle membrane | 428 | 1656 | 0.873 | 0.000 | 0.002 |
| GO:0012505 | Endomembrane system | 345 | 1414 | 0.825 | 0.029 | 0.039 |
| GO:0005739 | Mitochondrion | 305 | 1152 | 0.895 | 0.000 | 0.003 |
| GO:0098588 | Bounding membrane of organelle | 301 | 1183 | 0.860 | 0.005 | 0.012 |
|  | **Molecular function** |  |  |  |  |  |
| GO:0004842 | Ubiquitin-protein transferase activity | 37 | 132 | 0.975 | 0.049 | 0.050 |
| GO:0015171 | Amino acid transmembrane transporter act... | 23 | 64 | 1.250 | 0.006 | 0.048 |
| GO:0061630 | Ubiquitin protein ligase activity | 23 | 75 | 1.067 | 0.043 | 0.049 |
| GO:0042625 | Atpase activity, coupled to transmembran... | 23 | 76 | 1.053 | 0.050 | 0.050 |
| GO:0019200 | Carbohydrate kinase activity | 14 | 33 | 1.476 | 0.006 | 0.048 |
| GO:0000149 | SNARE binding | 14 | 36 | 1.353 | 0.014 | 0.049 |
| GO:0016408 | C-acyltransferase activity | 13 | 22 | 2.055 | 0.000 | 0.005 |
| GO:0044769 | Atpase activity, coupled to transmembran... | 13 | 30 | 1.507 | 0.006 | 0.048 |
| GO:0016846 | Carbon-sulfur lyase activity | 13 | 34 | 1.330 | 0.021 | 0.049 |
| GO:0004298 | Threonine-type endopeptidase activity | 12 | 19 | 2.197 | 0.000 | 0.005 |

**Supplementary information, Table S4.**

| **GO.ID** | **Term** | **DEGs size** | **Size** | **RF** | **P value** | **FDR** |
| --- | --- | --- | --- | --- | --- | --- |
|  | **Biological process** |  |  |  |  |  |
| GO:0044711 | Single-organism biosynthetic process | 487 | 1915 | 0.816 | 0.026 | 0.042 |
| GO:0051234 | Establishment of localization | 486 | 1911 | 0.816 | 0.026 | 0.042 |
| GO:0006810 | Transport | 479 | 1871 | 0.821 | 0.018 | 0.035 |
| GO:1902578 | Single-organism localization | 410 | 1614 | 0.815 | 0.044 | 0.045 |
| GO:0044765 | Single-organism transport | 385 | 1515 | 0.815 | 0.049 | 0.049 |
| GO:0051641 | Cellular localization | 287 | 1093 | 0.842 | 0.020 | 0.037 |
| GO:0051649 | Establishment of localization in cell | 246 | 902 | 0.875 | 0.005 | 0.022 |
| GO:0006396 | RNA processing | 234 | 842 | 0.891 | 0.002 | 0.019 |
| GO:0046907 | Intracellular transport | 212 | 754 | 0.902 | 0.002 | 0.018 |
| GO:0034660 | ncrna metabolic process | 197 | 706 | 0.895 | 0.004 | 0.022 |
|  | **Cellular component** |  |  |  |  |  |
| GO:0043227 | Membrane-bounded organelle | 1416 | 5627 | 0.802 | 0.000 | 0.002 |
| GO:0044422 | Organelle part | 987 | 3852 | 0.817 | 0.000 | 0.002 |
| GO:0044446 | Intracellular organelle part | 975 | 3788 | 0.821 | 0.000 | 0.002 |
| GO:0032991 | Macromolecular complex | 651 | 2535 | 0.819 | 0.006 | 0.015 |
| GO:0043234 | Protein complex | 486 | 1910 | 0.811 | 0.033 | 0.044 |
| GO:0098588 | Bounding membrane of organelle | 318 | 1183 | 0.857 | 0.005 | 0.014 |
| GO:0031975 | Envelope | 220 | 756 | 0.928 | 0.000 | 0.002 |
| GO:0031967 | Organelle envelope | 211 | 718 | 0.937 | 0.000 | 0.002 |
| GO:0005783 | Endoplasmic reticulum | 202 | 750 | 0.859 | 0.021 | 0.033 |
| GO:0030529 | Ribonucleoprotein complex | 199 | 711 | 0.892 | 0.004 | 0.013 |
|  | **Molecular function** |  |  |  |  |  |
| GO:0005198 | Structural molecule activity | 109 | 411 | 0.870 | 0.049 | 0.049 |
| GO:0098772 | Molecular function regulator | 85 | 309 | 0.903 | 0.034 | 0.042 |
| GO:0042625 | Atpase activity, coupled to transmembran... | 27 | 76 | 1.166 | 0.009 | 0.031 |
| GO:0019829 | Cation-transporting atpase activity | 24 | 65 | 1.212 | 0.008 | 0.031 |
| GO:0015171 | Amino acid transmembrane transporter act... | 23 | 64 | 1.179 | 0.013 | 0.031 |
| GO:0003743 | Translation initiation factor activity | 22 | 67 | 1.077 | 0.042 | 0.043 |
| GO:0009055 | Electron carrier activity | 21 | 63 | 1.094 | 0.039 | 0.042 |
| GO:0043021 | Ribonucleoprotein complex binding | 19 | 56 | 1.113 | 0.041 | 0.043 |
| GO:0019200 | Carbohydrate kinase activity | 16 | 33 | 1.591 | 0.001 | 0.017 |
| GO:0000149 | SNARE binding | 14 | 36 | 1.276 | 0.024 | 0.042 |

**Supplementary information, Table S5.**

| **GO.ID** | **Term** | **DEGs size** | **Size** | **RF** | **P value** | **FDR** |
| --- | --- | --- | --- | --- | --- | --- |
|  | **Biological process** |  |  |  |  |  |
| GO:0044237 | Cellular metabolic process | 93 | 6629 | 0.789 | 0.032 | 0.046 |
| GO:0006807 | Nitrogen compound metabolic process | 70 | 4721 | 0.834 | 0.031 | 0.046 |
| GO:0034641 | Cellular nitrogen compound metabolic pro... | 65 | 4141 | 0.883 | 0.011 | 0.045 |
| GO:0010467 | Gene expression | 46 | 2671 | 0.969 | 0.009 | 0.045 |
| GO:0071840 | Cellular component organization or bioge... | 45 | 2625 | 0.964 | 0.010 | 0.045 |
| GO:0016043 | Cellular component organization | 39 | 2262 | 0.970 | 0.017 | 0.045 |
| GO:0006996 | Organelle organization | 31 | 1493 | 1.168 | 0.003 | 0.022 |
| GO:1901566 | Organonitrogen compound biosynthetic pro... | 29 | 1692 | 0.964 | 0.045 | 0.049 |
| GO:1902589 | Single-organism organelle organization | 21 | 965 | 1.224 | 0.009 | 0.045 |
| GO:0006396 | RNA processing | 18 | 842 | 1.202 | 0.017 | 0.045 |
|  | **Cellular component** |  |  |  |  |  |
| GO:0005622 | Intracellular | 121 | 7901 | 0.856 | 0.012 | 0.021 |
| GO:0044424 | Intracellular part | 120 | 7818 | 0.858 | 0.012 | 0.021 |
| GO:0043229 | Intracellular organelle | 100 | 6044 | 0.925 | 0.004 | 0.014 |
| GO:0043226 | Organelle | 100 | 6109 | 0.915 | 0.006 | 0.015 |
| GO:0005737 | Cytoplasm | 98 | 6282 | 0.872 | 0.044 | 0.044 |
| GO:0043231 | Intracellular membrane-bounded organelle | 92 | 5567 | 0.924 | 0.009 | 0.019 |
| GO:0043227 | Membrane-bounded organelle | 92 | 5627 | 0.914 | 0.013 | 0.023 |
| GO:0044422 | Organelle part | 73 | 3852 | 1.059 | 0.001 | 0.004 |
| GO:0044446 | Intracellular organelle part | 72 | 3788 | 1.063 | 0.001 | 0.004 |
| GO:0043234 | Protein complex | 41 | 1910 | 1.200 | 0.002 | 0.011 |
|  | **Molecular function** |  |  |  |  |  |
| GO:0003723 | RNA binding | 21 | 1087 | 1.112 | 0.021 | 0.040 |
| GO:0005198 | Structural molecule activity | 11 | 411 | 1.540 | 0.011 | 0.037 |
| GO:0003735 | Structural constituent of ribosome | 10 | 243 | 2.368 | 0.001 | 0.032 |
| GO:0015077 | Monovalent inorganic cation transmembran... | 5 | 157 | 1.832 | 0.043 | 0.048 |
| GO:0016820 | Hydrolase activity, acting on acid anhyd... | 5 | 160 | 1.798 | 0.046 | 0.048 |
| GO:0042626 | Atpase activity, coupled to transmembran... | 5 | 160 | 1.798 | 0.046 | 0.048 |
| GO:0042625 | Atpase activity, coupled to transmembran... | 4 | 76 | 3.028 | 0.014 | 0.040 |
| GO:0015078 | Hydrogen ion transmembrane transporter a... | 4 | 85 | 2.708 | 0.020 | 0.040 |
| GO:0043566 | Structure-specific DNA binding | 4 | 112 | 2.055 | 0.048 | 0.048 |
| GO:0046933 | Proton-transporting ATP synthase activit... | 3 | 18 | 9.589 | 0.001 | 0.032 |

**Supplementary information, Table S6.**

| **GO.ID** | **Term** | **DEGs size** | **Size** | **RF** | **P value** | **FDR** |
| --- | --- | --- | --- | --- | --- | --- |
|  | Biological process |  |  |  |  |  |
| GO:0006996 | Organelle organization | 490 | 1493 | 0.795 | 0.046 | 0.049 |
| GO:0071702 | Organic substance transport | 379 | 1125 | 0.816 | 0.018 | 0.043 |
| GO:0008104 | Protein localization | 292 | 862 | 0.820 | 0.028 | 0.043 |
| GO:0046907 | Intracellular transport | 254 | 754 | 0.816 | 0.049 | 0.049 |
| GO:0045184 | Establishment of protein localization | 248 | 718 | 0.836 | 0.017 | 0.043 |
| GO:0015031 | Protein transport | 241 | 698 | 0.836 | 0.019 | 0.043 |
| GO:1902582 | Single-organism intracellular transport | 236 | 681 | 0.839 | 0.017 | 0.043 |
| GO:0055086 | Nucleobase-containing small molecule met... | 206 | 604 | 0.826 | 0.045 | 0.049 |
| GO:0042592 | Homeostatic process | 140 | 400 | 0.847 | 0.042 | 0.049 |
| GO:0044802 | Single-organism membrane organization | 116 | 327 | 0.859 | 0.041 | 0.049 |
|  | **Cellular component** |  |  |  |  |  |
| GO:0005623 | Cell | 2747 | 8775 | 0.753 | 0.023 | 0.037 |
| GO:0044464 | Cell part | 2742 | 8758 | 0.753 | 0.023 | 0.037 |
| GO:0005622 | Intracellular | 2514 | 7901 | 0.766 | 0.000 | 0.005 |
| GO:0044424 | Intracellular part | 2487 | 7818 | 0.765 | 0.000 | 0.005 |
| GO:0005737 | Cytoplasm | 2021 | 6282 | 0.774 | 0.000 | 0.005 |
| GO:0044422 | Organelle part | 1267 | 3852 | 0.791 | 0.001 | 0.005 |
| GO:0044446 | Intracellular organelle part | 1246 | 3788 | 0.791 | 0.001 | 0.005 |
| GO:0031090 | Organelle membrane | 568 | 1656 | 0.825 | 0.001 | 0.005 |
| GO:0012505 | Endomembrane system | 494 | 1414 | 0.841 | 0.000 | 0.005 |
| GO:0098588 | Bounding membrane of organelle | 416 | 1183 | 0.846 | 0.001 | 0.005 |
|  | **Molecular function** |  |  |  |  |  |
| GO:0050660 | Flavin adenine dinucleotide binding | 72 | 189 | 0.943 | 0.020 | 0.039 |
| GO:0016879 | Ligase activity, forming carbon-nitrogen... | 66 | 163 | 1.003 | 0.006 | 0.039 |
| GO:0015291 | Secondary active transmembrane transport... | 64 | 173 | 0.916 | 0.050 | 0.050 |
| GO:0016830 | Carbon-carbon lyase activity | 52 | 136 | 0.947 | 0.040 | 0.043 |
| GO:0015293 | Symporter activity | 39 | 91 | 1.061 | 0.010 | 0.039 |
| GO:0016831 | Carboxy-lyase activity | 39 | 91 | 1.061 | 0.010 | 0.039 |
| GO:0016667 | Oxidoreductase activity, acting on a sul... | 33 | 80 | 1.022 | 0.032 | 0.039 |
| GO:0031406 | Carboxylic acid binding | 29 | 65 | 1.105 | 0.014 | 0.039 |
| GO:0043177 | Organic acid binding | 29 | 65 | 1.105 | 0.014 | 0.039 |
| GO:0015295 | Solute: proton symporter activity | 21 | 47 | 1.107 | 0.032 | 0.039 |

**Supplementary information, Table S7.**

| **GO.ID** | **Term** | **DEGs size** | **Size** | **RF** | **P value** | **FDR** |
| --- | --- | --- | --- | --- | --- | --- |
|  | **Biological process** |  |  |  |  |  |
| GO:0044712 | Single-organism catabolic process | 49 | 753 | 0.968 | 0.031 | 0.050 |
| GO:0044282 | Small molecule catabolic process | 26 | 306 | 1.265 | 0.005 | 0.050 |
| GO:1901565 | Organonitrogen compound catabolic proces... | 23 | 273 | 1.254 | 0.009 | 0.050 |
| GO:0016054 | Organic acid catabolic process | 21 | 214 | 1.460 | 0.002 | 0.050 |
| GO:0046395 | Carboxylic acid catabolic process | 21 | 214 | 1.460 | 0.002 | 0.050 |
| GO:0019748 | Secondary metabolic process | 18 | 161 | 1.664 | 0.001 | 0.039 |
| GO:0044272 | Sulfur compound biosynthetic process | 17 | 206 | 1.228 | 0.028 | 0.050 |
| GO:0044550 | Secondary metabolite biosynthetic proces... | 16 | 124 | 1.920 | 0.000 | 0.029 |
| GO:0008202 | Steroid metabolic process | 12 | 141 | 1.267 | 0.049 | 0.050 |
| GO:0006694 | Steroid biosynthetic process | 11 | 103 | 1.589 | 0.013 | 0.050 |
|  | **Cellular component** |  |  |  |  |  |
| GO:0015630 | Microtubule cytoskeleton | 22 | 308 | 1.057 | 0.046 | 0.050 |
| GO:0032153 | Cell division site | 16 | 162 | 1.461 | 0.005 | 0.050 |
| GO:0098687 | Chromosomal region | 14 | 173 | 1.197 | 0.043 | 0.050 |
| GO:0032155 | Cell division site part | 7 | 69 | 1.501 | 0.050 | 0.050 |
| GO:0000779 | Condensed chromosome, centromeric region | 6 | 55 | 1.614 | 0.050 | 0.050 |
| GO:0016323 | Basolateral plasma membrane | 5 | 32 | 2.311 | 0.018 | 0.050 |
| GO:0005871 | Kinesin complex | 4 | 18 | 3.287 | 0.010 | 0.050 |
| GO:0005937 | Mating projection | 4 | 24 | 2.465 | 0.027 | 0.050 |
| GO:0033267 | Axon part | 4 | 25 | 2.367 | 0.031 | 0.050 |
| GO:0005968 | Rab-protein geranylgeranyltransferase co... | 3 | 4 | 11.094 | 0.000 | 0.016 |
|  | **Molecular function** |  |  |  |  |  |
| GO:0003824 | Catalytic activity | 375 | 6377 | 0.895 | 0.002 | 0.016 |
| GO:0016491 | Oxidoreductase activity | 87 | 1287 | 1.029 | 0.013 | 0.028 |
| GO:0003677 | DNA binding | 71 | 1062 | 1.018 | 0.030 | 0.039 |
| GO:0046914 | Transition metal ion binding | 60 | 848 | 1.077 | 0.016 | 0.028 |
| GO:0008270 | Zinc ion binding | 41 | 567 | 1.101 | 0.032 | 0.039 |
| GO:0048037 | Cofactor binding | 40 | 548 | 1.111 | 0.030 | 0.039 |
| GO:0001071 | Nucleic acid binding transcription facto... | 33 | 366 | 1.373 | 0.002 | 0.016 |
| GO:0003700 | Transcription factor activity, sequence-... | 33 | 366 | 1.373 | 0.002 | 0.016 |
| GO:0000981 | RNA polymerase II transcription factor a… | 27 | 267 | 1.540 | 0.001 | 0.016 |
| GO:0043565 | Sequence-specific DNA binding | 25 | 262 | 1.453 | 0.004 | 0.016 |

**Supplementary information, Table S8.**

| Pathway | Pfam | Genes | Description |
| --- | --- | --- | --- |
| Aminoacyl-  tRNA biosynthesis | PF03950 | Cluster-6812.0 | B8NQF7, Glutamyl-tRNA synthetase |
|  | PF00579 | Cluster-8450.0 | B8NCZ6, Tryptophanyl-tRNA synthetase, putative |
|  | PF00579 | Cluster-8933.0 | A0A0F0IH15, Uncharacterized protein |
|  | PF09334 | Cluster-10095.0 | A0A0F0IE09, Uncharacterized protein |
|  | PF09334 | Cluster-10095.1 | Q2UCY8, Uncharacterized protein |
|  | PF03129 | Cluster-5651.0 | A0A179GPZ6, Threonyl-tRNA synthetase |
|  | PF03129 | Cluster-6833.0 | B8NSH4, Glycyl-tRNA synthetase |
|  | PF03129 | Cluster-6873.0 | A0A179GVD1, Prolyl-tRNA synthetase 1 |
|  | PF03129 | Cluster-8882.0 | A0A0D9N8A5, tRNA synthetase class II core domain protein |
|  | PF03129 | Cluster-9310.0 | B8NSH4, Glycyl-tRNA synthetase |
|  | PF01409 | Cluster-6043.0 | I8IIQ1, Phenylalanyl-tRNA synthetase, beta subunit |
|  | PF01409 | Cluster-8311.0 | A0A179HGV5, Phenylalanyl-tRNA synthetase alpha subunit |
|  | PF00152 | Cluster-10092.0 | Q2URG4, Uncharacterized protein |
|  | PF00152 | Cluster-10092.1 | I8A678, Aspartyl-tRNA synthetase |
|  | PF00152 | Cluster-10092.3 | I8A678, Aspartyl-tRNA synthetase |
|  | PF00152 | Cluster-6909.0 | B8MZN2, Asparaginyl-tRNA synthetase Slm5, putative |
|  | PF01425 | Cluster-5114.0 | A0A179HEL8, Amidase signature |
|  | PF01425 | Cluster-5114.0 | A0A179HEL8, Amidase signature |
|  | PF01406 | Cluster-6615.0 | A0A179GR23, Leucyl-tRNA synthetase |
|  | PF01406 | Cluster-7403.0 | B8MXX8, Valyl-tRNA synthetase |
|  | PF01406 | Cluster-7598.0 | A0A0F0IEB8, Uncharacterized protein |
| Glycine, serine and threonine | PF00291 | Cluster-5300.1 | A0A0F0IAL3, Threonine synthase N terminu |
|  | PF00291 | Cluster-6965.0 | A0A0D9MPM9, Tryptophan synthase |
|  | PF00291 | Cluster-6965.1 | B8MZR1, Trytophan synthase alpha subunit, putative |
|  | PF00291 | Cluster-9734.0 | B8NBZ9, Cystathionine beta-synthase |
|  | PF00291 | Cluster-9734.0 | Q2TZA8, Uncharacterized protein |
|  | PF01053 | Cluster-6456.1 | Q2TYY9, Uncharacterized protein |
|  | PF03447 | Cluster-6168.0 | A0A0F0IGP7, Homoserine dehydrogenase |
|  | PF03447 | Cluster-9118.0 | A0A179GXS1, Homoserine dehydrogenase |
|  | PF00696 | Cluster-8789.0 | A0A179GKI4, Aspartokinase |
|  | PF00155 | Cluster-10322.0 | A0A179HIF1,5-aminolevulinate synthase |
|  | PF00155 | Cluster-10322.0 | A0A179H388,5-aminolevulinate synthase |
|  | PF00155 | Cluster-7924.0 | I7ZL47,5-aminolevulinate synthase |
|  | PF00155 | Cluster-7924.1 | I7ZL47,5-aminolevulinate synthase |
|  | PF02826 | Cluster-6258.0 | B8N947, D-lactate dehydrogenase, putative |
|  | PF01066 | Cluster-9667.0 | A0A0D9N7N4, CDP-diacylglycerol--serine O-phosphatidyltransferase |
|  | PF01066 | Cluster-9667.0 | A0A0E9NA45, Uncharacterized protein |
| Proteasome | PF00004 | Cluster-5912.0 | I8THB2,26S proteasome regulatory complex, ATPase RPT5 |
|  | PF00004 | Cluster-7309.0 | B8NNC8, Proteasome regulatory particle subunit Rpt5, putative |
|  | PF00004 | Cluster-8804.0 | A0A0D9MMT6, ATPase |
|  | PF01399 | Cluster-6019.0 | Q2UM28, Uncharacterized protein |
|  | PF01399 | Cluster-8912.1 | B8N2F8, Proteasome regulatory particle subunit |
|  | PF01399 | Cluster-9362.0 | B8N227, Proteasome regulatory particle subunit |
|  | PF01399 | Cluster-9368.0 | I7ZN25,26S proteasome regulatory complex, subunit RPN6/PSMD11 |
|  | PF01399 | Cluster-9692.0 | Q2U4H8, Uncharacterized protein |
|  | PF01399 | Cluster-9692.1 | A0A0D9MUM9, PCI domain protein |
|  | PF13519 | Cluster-8342.0 | B8N6T8,26S proteasome regulatory subunit S5A |
| Sulfur metabolism | PF01507 | Cluster-6254.0 | A0A0F0ILC3, Phosphoadenosine phosphosulfate reductase family protein |
|  | PF01507 | Cluster-6254.1 | A0A0D9MUE5, Phosphoadenosine phosphosulfate reductase family protein |
|  | PF00459 | Cluster-8383.1 | B8NPA7,3' |
|  | PF01053 | Cluster-9333.0 | A0A075X3C4, Cystathionine gamma-synthase |
| Fatty acid metabolism | PF00501 | Cluster-5733.0 | I8A0K3, Acyl-CoA synthetase |
|  | PF00108 | Cluster-7517.0 | Q2TZ23, Uncharacterized protein |
|  | PF00108 | Cluster-7517.1 | I8I934, Acetyl-CoA acetyltransferase |
|  | PF00108 | Cluster-8386.0 | I8U219,3-oxoacyl CoA thiolase |
|  | PF00107 | Cluster-9292.0 | A0A179HXF3, Alcohol dehydrogenase superfamily, zinc-containing |
|  | PF00107 | Cluster-9292.0 | A0A179HXF3, Alcohol dehydrogenase superfamily, zinc-containing |
| Fructose and mannose | PF00121 | Cluster-7978.1 | I8A3M4, Triosephosphate isomerase |
|  | PF01238 | Cluster-7638.0 | B8N4V5, Mannose-6-phosphate isomerase |
|  | PF01238 | Cluster-7703.0 | Q2UGT5, Uncharacterized protein |
| Ribosome biogenesis | PF01479 | Cluster-3749.0 | T0KCX0, Uncharacterized protein |
|  | PF01479 | Cluster-9199.1 | A0A179HM25, U3 small nucleolar ribonucleoprotein IMP3 |
|  | PF01479 | Cluster-9199.2 | A0A179HM25, U3 small nucleolar ribonucleoprotein IMP3 |
|  | PF01479 | Cluster-9199.2 | A0A179H0S5, U3 small nucleolar ribonucleoprotein IMP3 |
|  | PF01479 | Cluster-9199.3 | A0A179H0S5, U3 small nucleolar ribonucleoprotein IMP3 |
| Spliceosome | PF00076 | Cluster-10387.0 | A0A162CQ50, U2 snRNP auxiliary factor large subunit |

**Reference**

1. Kanehisa, M. & Goto, S. KEGG: Kyoto encyclopedia of genes and genomes. *Nucleic Acids Res.* **28,** 27–30 (2000).
